# The effect of biopolymer stabilisation on biostimulated or bioaugmented mine residue for potential technosol production

**DOI:** 10.1038/s41598-024-75840-0

**Published:** 2024-10-26

**Authors:** Joana B. Caldeira, António A. Correia, Rita Branco, Paula V. Morais

**Affiliations:** 1 Centre for Mechanical Engineering, Materials and Processes (CEMMPRE), ARISE, Department of Life Sciences, Universidade de Coimbra, 3000-456 Coimbra, Portugal; 2 Chemical Engineering and Renewable Resources for Sustainability (CERES), Department of Civil Engineering, Universidade de Coimbra, R. Luís Reis Santos, 3030-788 Coimbra, Portugal

**Keywords:** Bioleaching, Biopolymers, Metal recovery, Waste treatment, Sustainability, Microbiology, Environmental sciences, Engineering

## Abstract

Mine waste can be transformed into technosol as an ecological strategy. Despite its importance to soil functions, biological activity is often overlooked. Biopolymers can serve as innovative tools for bioremediation, facilitating chemical reactions and creating networks to encapsulate contaminants. This work aims to assess the use of bioleached and stabilised residues from a tungsten mine for technosol production. The first objective was to evaluate mine tailings for their bioleaching potential by biostimulation or bioaugmentation with strain *Diaphorobacter polyhydroxybutyrativorans* B2A2W2. The second was to evaluate the effect of Portland cement or biopolymers such as Carboxymethyl Cellulose (CMC) or Xanthan Gum (XG) on the stabilisation of bioleached residues. The impact of biopolymers on residues’ characteristics, such as metal leaching, number of cultivable microorganisms, compression strength and ecotoxicity was evaluated using flow systems. Over time, bioleached metallic elements decreased, except for iron (Fe). Biostimulated and stabilised residues exhibited similar trends; both CMC and cement showed low leaching rates and viable microorganisms in the same order (10^6^ CFU × ml^−1^). However, bioaugmented residue stabilised with XG showed 10^6^ CFU × ml^−1^ viable microorganisms and increased 2.2-fold Fe leaching than BA_Control. CMC addition to bioaugmented residue reduced 5.9-fold Fe leaching and increased 100-fold viable microorganisms. By utilising both biological and engineering approaches to characterise the technosol, this study contributes to advancing knowledge of technosol production. The residues biostimulated and stabilised with CMC produced a material useful for bio-applications, with low toxicity and metal leaching, useful for bio-applications. XG was the best stabiliser for geotechnical engineering applications, with improved compression strength. In conclusion, the study demonstrates the usefulness of biopolymer treatment for residues and emphasises the importance of selecting the appropriate biopolymer for the intended function of technosols.

**Supplementary Information:**

The online version contains supplementary material available at 10.1038/s41598-024-75840-0.

## Introduction

Mining activity employs energy-intensive engineering processes, such as deep soil excavation for ore exploration and landfill technologies. These processes generate large waste dams and piles of mine residues that negatively impact the environment^1^. Considering the huge socio-environmental risks associated with mine waste dams, a secondary exploration of these wastes to generate products with economic value should be considered. Different strategies have already been applied to reduce the environmental impact of mine tailings, resulting in a financial benefit. Mine waste often still contains significant valuable elements, and reprocessing these tailings can contribute to a circular economy and reduce the current European Union’s dependence on imported raw materials^2^. Several studies have demonstrated the possibility of using different tailings as a secondary source of critical or precious metals^3–5^. EU estimates that the total footprint of tailings is 28 billion tonnes, with a further 600 million tonnes being produced every year in Europe^6^. Adrianto and collaborators report that sulfidic copper tailings reprocessed can supply up to 2% of future European copper demand^7^. In Chile, 21 of the 744 tailings dams have economic potential for the reprocessing of metals such as copper, molybdenum and silver among others^8^. On the other hand, other studies have investigated the incorporation of additives and evaluated the possibility of improving the physicochemical properties of tailings (such as soil aggregation, soil water content, and resistance), to develop alternative and useful products^9,10^. However, the majority of the compounds employed in the modifications are very expensive and have limited applicability and action time^11^.

With the high number of tailing piles worldwide with both environmental risk and economic value, deciding the most appropriate procedures for managing this waste, whether to dispose of it or instead of utilising it as secondary raw materials is imperative. Thus, technosols may be considered a promising strategy to provide a new destination for re-mined or otherwise discarded wastes, thereby recovering the natural substrate for alternative purposes (e.g. agriculture and construction). The term ‘technosol’ describes human-handled soils containing different natural particles (sand, silt, clay) combined with other particles, including soil modifiers and waste materials (e.g. mine tailings, deconstruction-construction materials, industrial by-products). Furthermore, the World Reference Base^12^ stipulates that a technosol should contain at least 20% of artefacts, i.e., products created or substantially modified by humans from manufacturing processes and/or brought from depth to the surface by human activity (for example, industrial waste, oil products, bitumen, crude oil and mine spoil), have to be included. Technosols may contain some toxic substances^12^. In some cases, they are specifically constructed soils to solve a particular problem, such as metal pollution or acid mine drainage^13,14^. Traditionally, the development of technosols gives special emphasis on physicochemical characteristics and not on biological properties. Technosols are usually characterised by their tensile strength, compaction rate, or load-bearing capacity. The unconfined compression strength is a relevant parameter in real geotechnical work where the main actions are compression type^15^.

Technosols usually have low fertility, low organic matter content, and poor water-holding capacity. However, incorporating biopolymers into constructed soils can considerably improve the soil structure and properties (physical, chemical, and biological), enhancing nutrient availability and promoting plant growth. Biopolymers are high molecular weight molecules produced naturally by organisms with repeated sequences that can become reactive sites in chemical reactions by binding metals or soil particles or both (forming complexes with geopolymers). Biopolymers were already used in some industries to act as road construction binders and strengthen soil^16,17^. Biopolymers are potentially more effective at increasing particle interaction (such as chitosan biopolymer) and reducing erosion in soils than non-biological polymers^18–20^. Biopolymers such as starch and xanthan gum (XG) hydrogels^21^ can also help bind soil particles together, blocking soil pores and enhancing soil compaction. In previous studies, XG was able to chelate cadmium (Cd), copper (Cu), lead (Pb), and zinc (Zn) from heavy metal-contaminated soil^22^, a biochemical composite with carboxymethyl cellulose (CMC) promoted the release of Pb from a Pb-contaminated soil^23^. Moreover, applying biopolymers into soils can activate soil biological activity since it provides an extra source of organic matter beneficial to soil organisms. These biopolymers were described to promote better results with concentrations of 1-1.5% w/w of XG and 1% w/w of CMC to the soil mass^24,25^.

Mine tailings from a Portuguese tungsten (W) mine (Panasqueira mine) were shown to contain a microbiome consisting of *Acinetobacter*, *Bacillus*, *Cellulomonas*, *Pseudomonas*, *Streptococcus* and *Rothia* as the main bacterial genera^26^. Furthermore, tailings from different basins of this particular mine, constructed and used at different times throughout the mine’s operation, exhibited different metal concentrations in the tailings and different microbiome compositions^26^.

Several microorganisms from this mine were isolated and characterised, with particular consideration to their strategies to cope with different metals. The technology to be used to remediate mine tailings, whether in general or in this specific case, must consider the activity of microorganisms^5,27–30^. The tailings from the Panasqueira mine contain lower concentrations of metals that are considered critical by the European Commission^26,31^.

A study conducted in China showed that due to inefficient mining technologies, hundreds of kilotons of W are lost in the tailings^32^. These findings prove a need to explore new recycling recovery technologies to remove metals from mine tailings. One promising approach for the reuse of Panasqueira tailings is to leach low-value components, re-extract the enriched relevant critical metals and dispose of the residues^5^. The main focus of this study is to assess the use of bioleached and stabilised residues from a tungsten mine for technosol production, using a two-step process for treating and utilising the high metal content of the Panasqueira mine tailings. These materials have high availability and potential for constructing new soils. The first specific objective was to assess the bioleaching potential of Panasqueira mine tailings through the utilisation of biostimulation or bioaugmentation with an autochthonous strain *Diaphorobacter polyhydroxybutyrativorans* B2A2W2. This process is intended to determine the time required to start leaching metals and the total amount of metals leached after activation with biotreatment. The second specific objective was to assess the impact of Portland cement or biopolymers, including CMC and XG, on the stabilisation of bioleached residues and evaluate the effect on the promotion of technosol biological activity. These stabilisations’ influence on the residues’ characteristics was assessed through metal leaching, the number of cultivable microorganisms, compression strength, and ecotoxicity using laboratory column flow systems.

## Results

### Characterisation of Panasqueira mine residues and stabilisers

The X-ray fluorescence (XRF) analysis revealed that the waste (original residue - OR) contained the following major metals: silicon (Si) (38.7%), aluminium (Al) (24.2%), iron (Fe) (16.4%), potassium (K) (10.8%), lead (Pb) (4.2%), arsenic (As) (1.4%), titanium (Ti) (1.4%), calcium (Ca) (0.9%), sulfur (S) (0.4%) and tungsten (W) (0.3%). The soil has a porosity of 52% and an average water content of 19%. The residue OR was characterised by grain size composition, with 61.6% of particles classified as sand, 35.7% as silt and 2.7% as clay. The residue characterisation revealed that the maximum diameter of 90% of the particles was 223.12 μm, the biological oxygen demand was 0.15 g l^−1^, the total organic carbon in sediments was 0.65%, and the net acid generation potential was 7.84 kg H_2_SO_4_/t^26^.

The microbiological characterisation showed that the core microbiome of the mine residue comprises members of the family and six genera^26^. The strains of the family *Comamonadaceae* were detected in the samples characterised from this mine tailing^26^. The strain *Diaphorobacter* B2A2W2 is a member of the *Comamonadaceae* family and was isolated from the tailings deposited in basin 2 using the Reasoner’s 2A (R2A) medium^5^. This strain was previously shown to resist 100 mM W, 100 mM Mo and 2 mM Cu. The strain is also shown to cope with a pH of 2.5–3.5^5^.

The stabilisers used were Portland cement, CMC and XG. Portland cement had a 2.16% magnesium (Mg) oxide concentration. CMC comprises a chemical compound with the formula C_28_H_30_Na_8_O_27_ and an average molecular weight of 700,000 g mol^−1^. XG is a polysaccharide (anionic) with a molecular weight greater than 1,000,000 g mol^−1^ and composed of a backbone β-(1→4)-d-glucopyranose glucan and (1→3)-α-d-mannopyranose-(2→1)-β-d-glucuronic acid-(4→1)-β-d-mannopyranose as side chains on alternating residues.

### Time-dependent bioleaching profile of the residue

The impact of the bacterial strain B2A2W2 addition to the Panasqueira mine residue (bioaugmentation—BA) and the stimulation of the residue’s microbiome (biostimulation—BS) was compared by evaluating the metal content of the leachates in both experiments and the produced bioleached residues.

The pH of the residue bioaugmented and biostimulated did not change by more than 0.1 over time (Table 1). The average pH was approximately 2.6 for both conditions. Cultivable bacteria were only detected by colony-forming units (CFU) in the leachate from the bioaugmented system, CFU × ml^−1^ ranging from 1.7 to 8.7 × 10^1^ (Table 1). Even though microorganisms were not detected in the leachate after the first biostimulation cycle, this does not imply that there are no living organisms with the capacity to multiply in the residue. It can be related to a low number of microorganisms in the residue in the present cycle.

**Table 1:** pH values and microorganisms’ CFU obtained at different time points (0, 2, 5 and 7 days) of the leachate from bioleached residue.

		(days)	0	2	5	7
BS	Biostimulation	pH	2.64 ± 0.21	2.48 ± 0.11	2.60 ± 0.12	2.51 ± 0.11
		CFU × ml^−1^	0.0 × 10^0^ ± 0.0 × 10^0^	0.0 × 10^0^ ± 0.0 × 10^0^	0.0 × 10^0^ ± 0.0 × 10^0^	0.0 × 10^0^ ± 0.0 × 10^0^
BA	Bioaugmentation	pH	2.66 ± 0.14	2.58 ± 0.11	2.62 ± 0.13	2.57 ± 0.15
		CFU × ml^−1^	1.7 × 10^0^ ± 1.1 × 10^0^	2.5 × 10^0^ ± 1.3 × 10^0^	1.1 × 10^1^ ± 5.2 × 10^0^	8.7 × 10^1^ ± 3.0 × 10^1^

The time-dependent analysis of the concentration of the metals in leachate revealed that, for both bioleaching processes, the majority of detected elements exhibited lower concentrations at the final incubation time (7 days) in comparison to the initial time (negative values of absolute time-dependent variation) (Table 2). Of the elements exhibiting negative variation, only S exhibited concentrations above hundreds of mg × l^−1^ in the leachate. In contrast, the element Cu showed concentrations in the tens of mg × l^−1^.

The metals present in the leachates that demonstrated a positive variation over time were Fe and selenium (Se), meaning they increased the amount leached with incubation time. However, only Fe concentration increased considerably with incubation time, from 10.4 ± 7.5 mg × l^−1^ to 93.2 ± 37.2 mg × l^−1^ in biostimulation and from 11.4 ± 6.4 mg × l^−1^ to 42.9 ± 5.9 mg × l^−1^ in bioaugmentation assays. Consequently, the biostimulation promoted significantly higher Fe mobilisation (2.2-fold) than the bioaugmentation. The other metals were most leached in the first days of incubation. Furthermore, the leachates analysed did not contain any detectable levels of W and manganese (Mn).

**Table 2:** First bioleaching event.

		Metal	Metal concentration in the leachate (mg × l^−1^)		Absolute time-dependent variation (mg × l^−1^)	Absolute time-dependent variation per gram of soil (mg × l^−1^ × g^−1^)
			1st day	7nd day		
BS	Biostimulation	Al	13.17 ± 3.33	8.93 ± 3.88	− 4.24 ± 2.51	− 0.03 ± 0.02
		Cu	34.41 ± 15.03	11.67 ± 1.98	− 22.74 ± 7.58	− 0.14 ± 0.05
		Fe	10.42 ± 7.47	93.21 ± 37.16	82.79 ± 18.95	0.52 ± 0.06
		K	13.23 ± 4.65	3.87 ± 2.82	− 9.36 ± 2.72	− 0.06 ± 0.02
		Mg	10.67 ± 0.59	1.32 ± 1.13	− 9.35 ± 0.63	− 0.06 ± 0.00
		Zn	18.35 ± 3.07	13.68 ± 3.96	− 4.67 ± 2.51	0.03 ± 0.02
		Se	0.41 ± 0.41	0.62 ± 0.62	0.21 ± 0.37	0.00 ± 0.00
		P	0.56 ± 0.56	0.93 ± 0.93	0.37 ± 0.54	0.00 ± 0.00
		S	269.62 ± 46.67	172.37 ± 11.12	− 97.25 ± 23.99	− 0.61 ± 0.15
		Si	30.52 ± 0.23	26.87 ± 0.03	− 3.65 ± 0.12	− 0.02 ± 0.00
BA	Bioaugmentation	Al	14.85 ± 3.94	8.41 ± 1.91	− 6.44 ± 2.19	− 0.04 ± 0.01
		Cu	34.65 ± 14.83	10.69 ± 0.58	− 23.96 ± 7.42	− 0.15 ± 0.05
		Fe	11.39 ± 6.44	42.87 ± 5.95	31.48 ± 4.38	0.20 ± 0.03
		K	7.14 ± 2.13	3.02 ± 1.16	− 4.12 ± 1.21	− 0.03 ± 0.01
		Mg	11.28 ± 0.57	1.62 ± 1.62	− 9.66 ± 0.86	− 0.06 ± 0.01
		Zn	15.88 ± 4.43	7.35 ± 3.33	− 8.54 ± 2.77	0.05 ± 0.02
		Se	0.00 ± 0.00	0.53 ± 0.53	0.53 ± 0.26	0.00 ± 0.00
		P	1.75 ± 1.75	1.46 ± 0.19	− 0.29 ± 0.88	0.00 ± 0.01
		S	296.70 ± 50.49	144.20 ± 9.17	− 152.49 ± 25.66	− 0.95 ± 0.16
		Si	31.43 ± 0.44	26.57 ± 1.44	− 4.87 ± 0.75	− 0.03 ± 0.01

Metal concentration (mg × l^−1^) values and values of absolute time-dependent variation (mg × l^−1^) of metals in the leachate of samples from the BS and BA experiments.

### Effect of stabiliser addition on the leachate composition from the original residue

The pH of the leachate of the original residue varied from 2.6 to 2.2 with the leaching process. The addition of the stabilisers to the residue increased the pH, particularly in the case of cement and CMC. In all tested conditions, there was a slight decrease in pH over time (Table 3).

**Table 3:** The mean pH values and microorganisms’ CFU of the leachate of the original residue obtained at different time points (0, 2, 5 and 7 days), without biotreatment, but stabilised with different compounds.

	(days)	0	2	5	7
OR_Control	pH	2.55 ± 0.01	2.26 ± 0.01	2.51 ± 0.03	2.20 ± 0.03
	CFU × ml^−1^	1.5 × 10^1^ ± 5.0 × 10^0^	0.0 × 10^0^ ± 0.0 × 10^0^	0.0 × 10^0^ ± 0.0 × 10^0^	0.0 × 10^0^ ± 0.0 × 10^0^
OR_Ref	pH	6.40 ± 0.20	6.61 ± 0.23	6.07 ± 0.17	5.87 ± 0.34
	CFU × ml^−1^	1.2 × 10^3^ ± 2.1 × 10^2^	1.7 × 10^4^ ± 3.5 × 10^3^	7.8 × 10^5^ ± 9.5 × 10^4^	3.9 × 10^5^ ± 2.9 × 10^4^
OR_CMC	pH	3.98 ± 0.19	3.73 ± 0.21	3.70 ± 0.21	3.49 ± 0.19
	CFU × ml^−1^	1.6 × 10^3^ ± 6.5 × 10^2^	4.5 × 10^3^ ± 2.0 × 10^2^	2.2 × 10^3^ ± 3.2 × 10^2^	1.3 × 10^6^ ± 2.4 × 10^5^
OR_XG	pH	2.99 ± 0.03	2.73 ± 0.00	2.81 ± 0.02	2.64 ± 0.09
	CFU × ml^−1^	0.0 × 10^0^ ± 0.0 × 10^0^	0.0 × 10^0^ ± 0.0 × 10^0^	0.0 × 10^0^ ± 0.0 × 10^0^	0.0 × 10^0^ ± 0.0 × 10^0^

The residue control (OR_Control) had a mean of 15 CFU × ml^−1^ in the leachate at the beginning of the assay. After further incubation time, no cultivable bacteria were found. In contrast, residue stabilised with cement (OR_Ref) and CMC (OR_CMC) exhibited other behaviour, with CFU × ml^−1^ increasing from 10^3^ to 10^5^ or 10^6^, respectively. The addition of XG resulted in no detection of cultivable bacteria (Table 3).

**Fig. 1:**
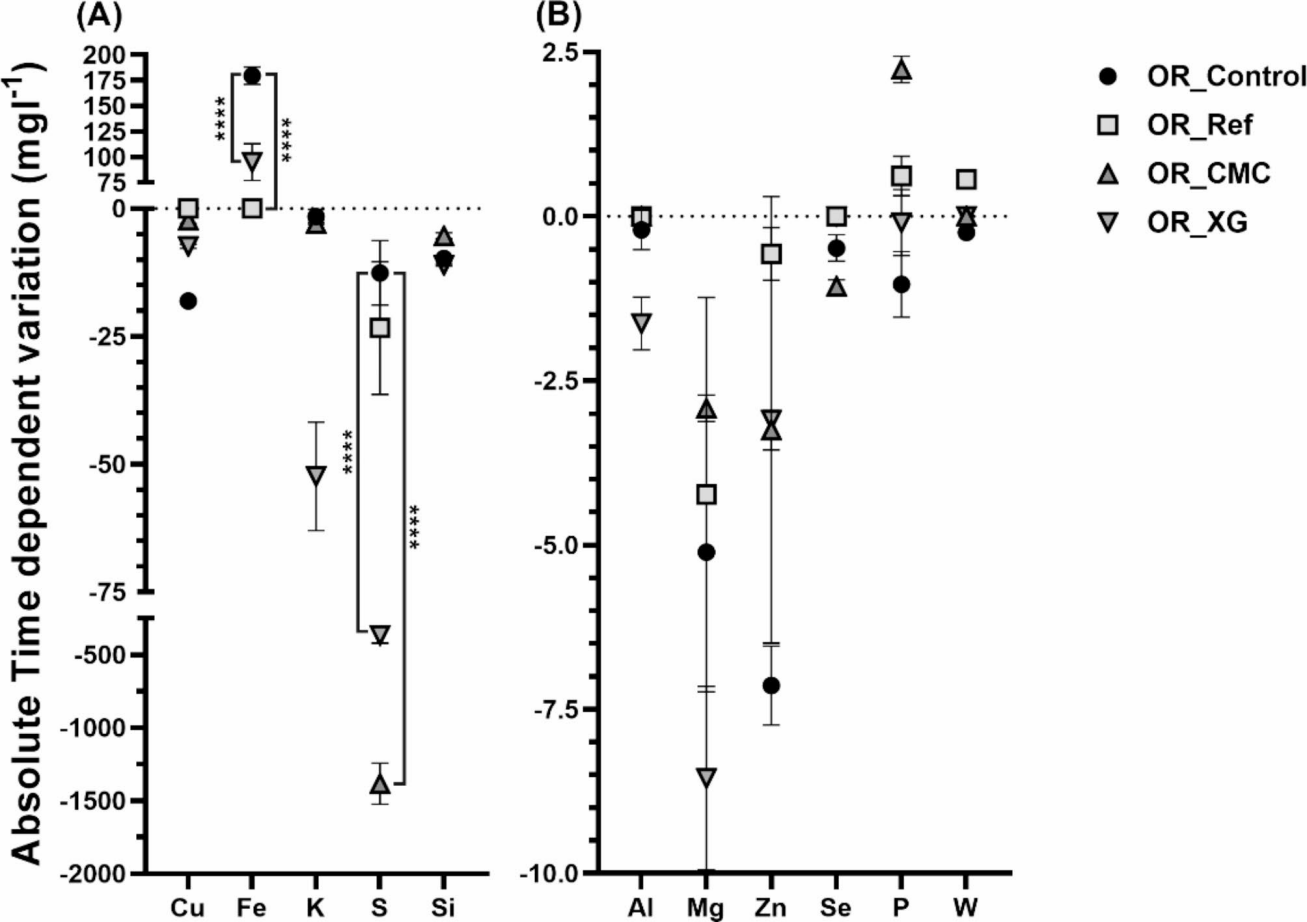
Absolute time-dependent variation of metal concentration (mg × l^−1^) in the leachates of residues, stabilised or not, OR_Control, OR_Ref, OR_CMC and OR_XG. Metals with high variation (> 10 m mg × l^−1^) (**A**) and low variation (< 10 mg × l^−1^) (**B**). The variation was calculated from the difference in metal content between 7 and 0 d. The data shown are the mean values of two independent experiments (± standard deviations). ****Significantly different, p < 0.0001.

Leachate from the control assay (without stabilisers) showed a positive metal concentration variation only to Fe, with absolute concentrations from 8.2 ± 0.0 mg × l^−1^ to 187.6 ± 17.0 mg × l^−1^ (Fig. 1 and S1). This means that the only metal that increased the amount leached with time was Fe. All the other detected metals exhibited a decrease over time, with the highest metal decreases observed for Cu and Zn, which varied from 27.6 ± 0.8 mg × l^−1^ at day 0 to 9.6 ± 0.2 mg × l^−1^ at day 7, and 12.9 ± 0.8 mg × l^−1^ at day 0 to 5.8 ± 0.7 mg × l^−1^ at day 7, respectively (Figs. 1 and S1). In general, the addition of XG to the residue did not affect the metal mobilisation, as similar metal contents were detected when comparing the metals leached from non-stabilised control residue and xanthan stabilised residue (OR_Control and OR_XG), except for S and K. On the other hand, the stabilisation with cement or CMC affected the leaching profile similarly. The stabilisation with cement and CMC leads to a reduction in the metal concentration of the leachates. Notably, both additives decreased the Fe leaching values to approximately 0 mg × l^−1^, while both led to the mobilisation of phosphorus (P) to the leachate, with a positive trend with time. Moreover, CMC addition significantly affected the S mobilisation.

### Effect of stabiliser addition on leachate composition from the bioleached residue

A comparison of the biological activity in the leachates from residues non-treated with stabilisers from the first and second leaching cycles (Table 1—BS and BA and Table 4—BS_Control and BA_Control, respectively) indicates that the second leaching cycle promoted an increase in the number of microorganisms in the leachate, with CFU × ml^−1^ values rising from 10^3^ to 10^4^ (biostimulation) or 10^5^ (bioaugmentation). The mean pH of the leachates measured in those obtained in the first cycle was identical for both experiments (2.6), but they exhibited a slight increase during the second cycle (2.8 for biostimulation and 3.1 for bioaugmentation).

**Fig. 2:**
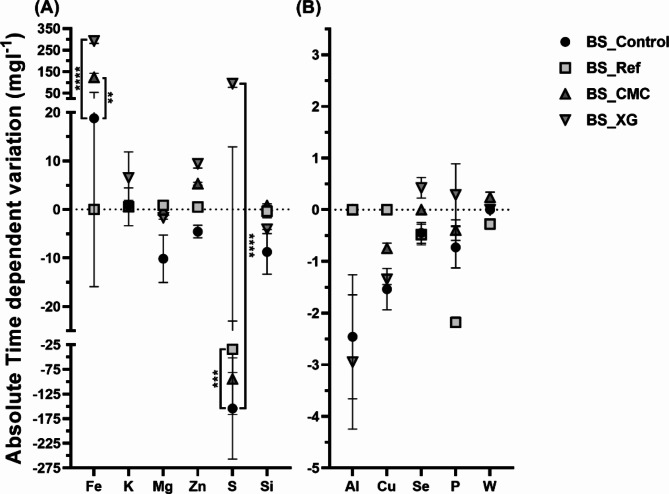
Second leaching cycle. Absolute time-dependent variation of metal concentration (mg × l^−1^) in the leachates of biostimulated residues, stabilised or not, BS_Control, BS_Ref, BS_CMC and BS_XG. Metals with high variation (> 5 mg × l^−1^) (**A**) and low variation (< 5 mg × l^−1^) (**B**). The variation was calculated from the difference in metal content between 7 and 0 d. The data shown are the mean values of two independent experiments (± standard deviations). **^,^***^,^****Significantly different, p < 0.01, p < 0.001 and p < 0.0001, respectively.

**Fig. 3:**
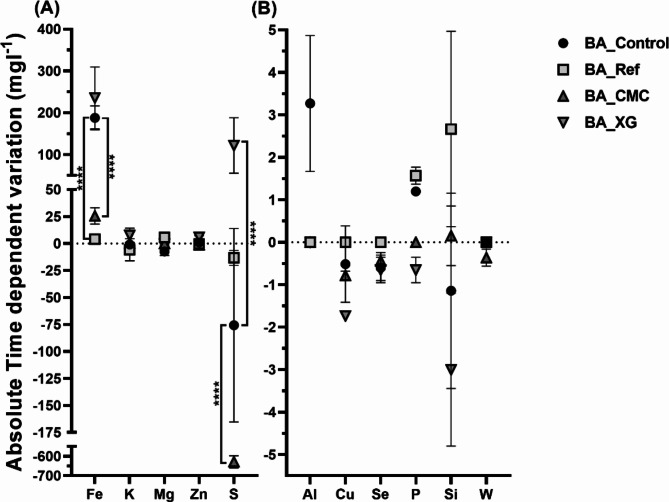
Second leaching cycle. Absolute time-dependent variation of metal concentration (mg × l^−1^) in the leachates of bioaugmented residues, stabilised or not, BA_Control, BA_Ref, BA_CMC and BA_XG. Metals with high variation (> 5 mg × l^−1^) (**A**) and low variation (< 5 mg × l^−1^) (**B**). The variation was calculated from the difference in metal content between 7 and 0 d. The data shown are the mean values of two independent experiments (± standard deviations). ****Significantly different, p < 0.0001.

In the second leaching cycle, the non-stabilised control samples (BS_Control and BA_Control) showed a similar pattern of metals leached as in the first cycle (BS and BA, respectively). The leaching levels followed the order S > Fe > Si > Zn > Mg (Figs. 2 and 3, S2 and S3). However, in this second cycle, the Cu amount was 10-fold lower (BS_Control and BA_Control, the absolute concentrations ranged from 1.3 ± 0.4 mg × l^−1^ and 3.3 ± 0.8 mg × l^−1^ Cu) than in the first cycle (the maximum and minimum Cu concentrations for BS and BA ranged from 10.7 ± 0.6 mg × l^−1^ and 34.7 ± 14.8 mg × l^−1^ Cu) (Figs. 2 and 3, respectively, and Table 2). Considering the metal concentration in the leachates with residues not stabilised, the metal leaching process might continue over time.

The final pH of the leachates from residues bioleached and stabilised was higher than the pH of residues bioleached and not stabilised. The highest pH was registered for the residues stabilised with cement (6.3 and 5.6), while the other additives resulted in pH < 4 (Table 4).

**Table 4:** Second leaching cycle.

	(days)	0	2	5	7
BS_Control	pH	2.99 ± 0.32	2.74 ± 0.17	2.66 ± 0.13	2.83 ± 0.13
	CFU × ml^−1^	1.3 × 10^3^ ± 1.7 × 10^2^	3.2 × 10^3^ ± 4.6 × 10^2^	4.7 × 10^3^ ± 4.2 × 10^2^	8.2 × 10^4^ ± 6.3 × 10^3^
BS_Ref	pH	6.83 ± 0.02	6.37 ± 0.08	6.01 ± 0.16	6.28 ± 0.09
	CFU × ml^−1^	2.4 × 10^6^ ± 5.5 × 10^5^	3.0 × 10^5^ ± 2.8 × 10^4^	1.1 × 10^6^ ± 1.4 × 10^5^	1.9 × 10^6^ ± 8.0 × 10^5^
BS_CMC	pH	3.57 ± 0.13	3.15 ± 0.27	3.23 ± 0.31	3.13 ± 0.02
	CFU × ml^−1^	4.4 × 10^4^ ± 4.5 × 10^3^	1.5 × 10^5^ ± 2.9 × 10^4^	9.6 × 10^5^ ± 3.5 × 10^4^	3.2 × 10^6^ ± 5.9 × 10^5^
BS_XG	pH	3.00 ± 0.02	3.20 ± 0.01	3.41 ± 0.07	2.99 ± 0.00
	CFU × ml^−1^	1.7 × 10^5^ ± 1.9 × 10^4^	2.2 × 10^5^ ± 1.3 × 10^4^	1.9 × 10^5^ ± 2.5 × 10^4^	2.0 × 10^5^ ± 4.5 × 10^4^
BA_Control	pH	3.30 ± 0.54	3.24 ± 0.44	3.00 ± 0.28	3.05 ± 0.20
	CFU × ml^−1^	1.8 × 10^3^ ± 9.5 × 10^2^	1.1 × 10^4^ ± 2.5 × 10^3^	3.9 × 10^4^ ± 5.9 × 10^3^	1.7 × 10^5^ ± 4.8 × 10^4^
BA_Ref	pH	5.94 ± 0.55	5.77 ± 1.06	5.59 ± 0.83	5.60 ± 1.17
	CFU × ml^−1^	4.6 × 10^5^ ± 3.5 × 10^4^	1.6 × 10^6^ ± 5.7 × 10^5^	1.4 × 10^6^ ± 3.4 × 10^5^	1.8 × 10^6^ ± 7.5 × 10^5^
BA_CMC	pH	3.93 ± 0.19	3.51 ± 0.09	3.74 ± 0.00	3.65 ± 0.14
	CFU × ml^−1^	8.7 × 10^4^ ± 4.0 × 10^3^	2.6 × 10^5^ ± 2.1 × 10^4^	1.4 × 10^7^ ± 2.0 × 10^6^	4.0 × 10^7^ ± 1.9 × 10^6^
BA_XG	pH	3.06 ± 0.00	3.21 ± 0.01	3.36 ± 0.12	3.30 ± 0.02
	CFU × ml^−1^	1.8 × 10^5^ ± 3.5 × 10^4^	2.5 × 10^6^ ± 5.0 × 10^4^	1.7 × 10^6^ ± 2.5 × 10^5^	5.9 × 10^6^ ± 8.5 × 10^5^

pH values of leachates from residues previously bioleached and stabilised with different compounds (second leaching cycle) at different time points (0, 2, 5 and 7 days).

As mentioned before for the non-stabilised samples, regardless of the stabiliser added, the number of microorganisms present in leachates from the second cycle was higher than in the first cycle (Tables 1 and 4). The CFU × ml^−1^ detected increased along the incubation time, achieving the maximum value of 10^7^ CFU × ml^−1^ with CMC stabilisation (Table 4).

The residues bioleached by biostimulation and then stabilised with cement (BS_Ref), CMC (BS_CMC) or XG (BS_XG) were evaluated for the possible bioleaching of the metals previously detected in the first cycle (Figs. 2 and S2). A comparison of the leachate composition in metals content of BS_Ref with BS_Control revealed that the only statistically significant difference was observed in the S leaching in the second cycle (absolute values ranging from 297.8 ± 204.8 mg × l^−1^ to 143.6 ± 0.4 mg × l^−1^ S in BS_Control and from 465.4 ± 49.7 mg × l^−1^ to 431.2 ± 80.0 mg × l^−1^ S in BS_Ref). Nevertheless, differences were observed for Al, Cu and Fe. In BS_Ref, no leaching was observed for those metals, whereas a second cycle of leaching (BS_Control) led to the time-dependent variation observed which was a decrease in the Al and Cu leached (− 2.5 ± 1.2 mg × l^−1^ Al, − 1.5 ± 0.4 mg × l^−1^ Cu) and an increase of Fe (18.8 ± 34.7 mg × l^−1^ Fe). The CMC stabilisation of bioleached residue affected considerably only the leaching of Fe, which increased approximately 6.5-fold (of absolute time-dependent variation) compared with the control condition. However, the stabilisation with XG increased both Fe and S leaching (absolute values ranging from 212.9 ± 15.5 mg × l^−1^ to 505.7 ± 17.7 mg × l^−1^ Fe and 323.9 ± 32.3 mg × l^−1^ to 418.9 ± 14.6 mg×l^−1^ S).

The results showed that Portland cement and CMC had similar abilities to reduce the metal concentration in leachates.

The residue bioleached by bioaugmentation (BA_Control) without stabilisation showed increased Fe leached over time (Figs. 3 and S3). This bioleached residue was stabilised with cement (BA_Ref), CMC (BA_CMC) and XG (BA_XG). The amount of Fe measured in the leachate after cement stabilisation was significantly lower than in the control (46.2-fold lower absolute time-dependent variation) (Figs. 3 and S3). The stabilisation with XG (BA_XG) promoted an increase of Fe, Zn and S leached with time (positive time-dependent variation of absolute values ranging from 294.6 ± 46.7 mg×l^−1^ Fe to 530.0 ± 140.4 mg×l^−1^ Fe; from 7.7 ± 0.6 mg × l^−1^ to 13.6 ± 2.7 mg × l^−1^ Zn; and from 276.7 ± 6.2 mg × l^−1^ to 398.2 ± 132.9 mg × l^−1^ S). Conversely, the CMC (BA_CMC) addition led to a significant reduction in the amount the metals leached, particularly S (8.3-fold lower in absolute time-dependent variation) and Fe (7.3-fold lower in absolute time-dependent variation) when compared with the residue not stabilised (BA_Control). A comparison between the stabilised residues BA_Ref (with cement) and BA_XG revealed that cement promotes the increase in the leachate’s Si and P over time, while XG promotes a decrease of these metals leaching. The Fe leaching was particularly promoted by biotreatments and by the application of XG.

**Fig. 4:**
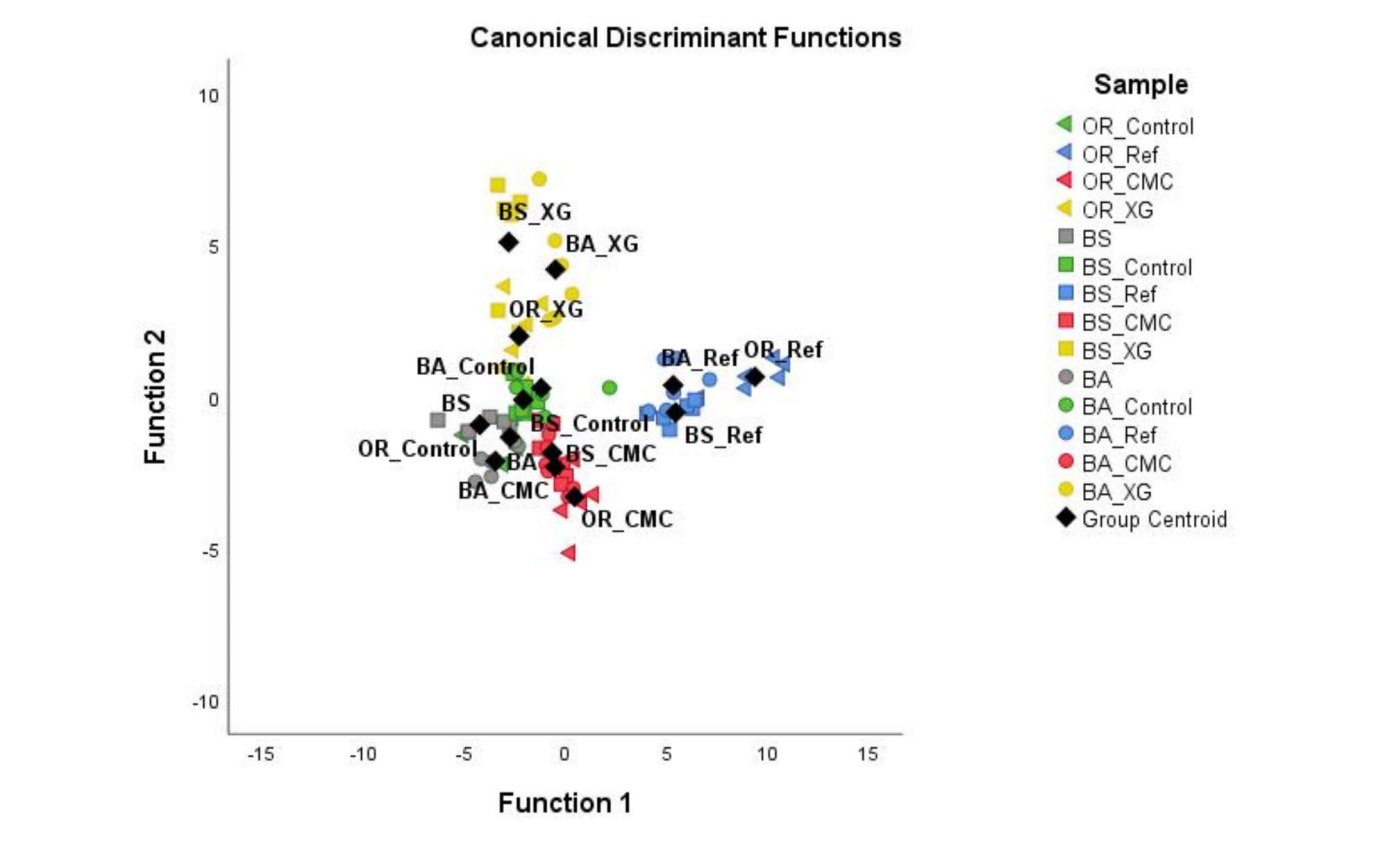
Leached residues discriminant function analysis based on the concentration of the metals in the leachate obtained at different time points (0, 2 and 7 days).

Discriminant Function Analysis (DFA) is a statistical analysis that classifies samples according to their respective sample groups. This analysis was performed considering the leached metal concentrations of all residue samples at different time points (Fig. 4). The two Discriminant Functions explain, respectively, 49.0% (function 1) and 18.1% (function 2) of the variance (i.e., in Fig. 4 and 67.1% of the variance is explained). Function 1 is highly impacted by the concentration of Mg in the leachates (1.197) and negatively by the concentration of Si (− 1.114) and Zn (− 0.504). On the other hand, Function 2 is highly impacted by the concentration of Fe in the leachates (0.711) and K (0.673) and negatively by the concentration of S (− 0.573).

Figure 4 shows that Control samples of the residue (leaching profile of the residue analysed without stabiliser addition) are closer to each other and to the zero of both functions, with no clear tendency. The statistical analysis did not differentiate the samples based on the biotreatment. Instead, the samples were grouped based on the stabiliser used on them. The stabilised residues with cement (Ref samples) were separated from the other samples on Function 1, which seems related to a higher concentration of Mg leached by these samples. The residue samples stabilised with XG (XG samples) were separated from the other samples in the analysis due to the impact of Function 2 (high concentrations of Fe leached). The CMC-stabilised residue samples were grouped closer to the non-stabilised samples. This analysis demonstrates that stabilisers exerted different effects on the residues.

### Mechanical characterisation of residue stabilised

**Fig. 5:**
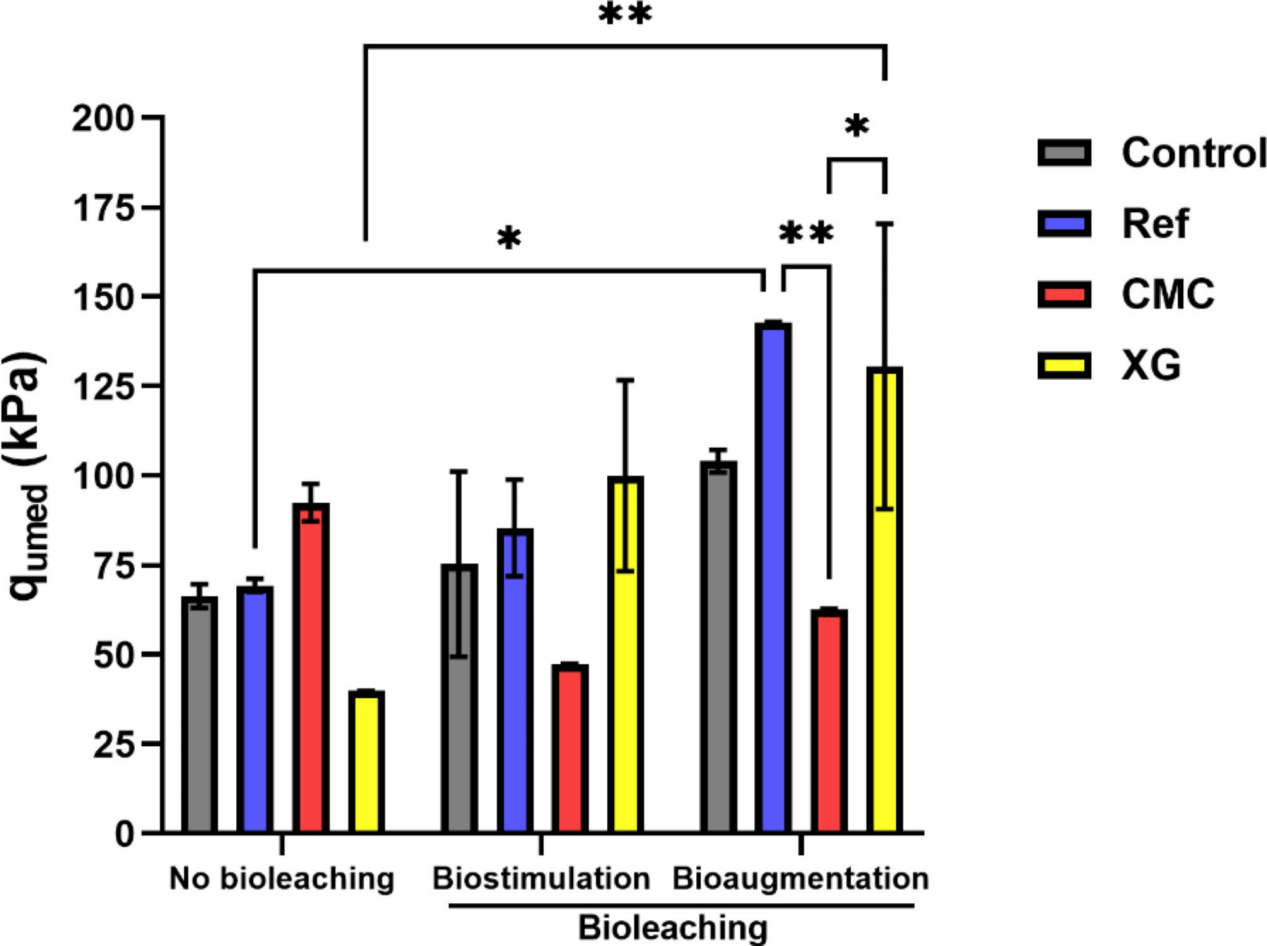
Stabilised residue strength determined by UCS test. The data shown are the mean values of two independent experiments (± standard deviations). *^,^**Significantly different, p < 0.05 and p < 0.01, respectively.

The unconfined compressive strength (UCS) test, also known as the Uniaxial Compression Strength test, is a commonly employed method for characterising mechanical proprieties of soils. Its simplicity and rapid execution allow the assessment of stress-strain curves under monotonic compression. The stabilisation of the residues determined by the UCS test was compared to evaluate the existence of differences statistically relevant to the strength of the residues leached (Fig. 5). Again, residues stabilised with cement were used as a reference for each experiment. This comparison was done within each experiment type (comparing the results of different stabilisers) and between experiments (comparing the results of the same stabiliser). Within the experiment types, only the bioaugmentation treatment showed statistically relevant differences in the stabilisation of the residue with CMC (62.76 kPa) and the stabilisation with cement (Ref) and XG (142.82 kPa and 130.48 kPa, respectively). Comparing different experiments with the same stabiliser, it was observed that, when the residue was stabilised with XG, there were significant differences between the residue treated without biotreatment (39.84 kPa) and with bioaugmentation (130.48 kPa). The same behaviour was observed when the cement was used as a stabiliser (69.35 kPa without biotreatment and 142.82 kPa with the bioaugmentation).

The compressive strength was all higher than the obtained for samples not leached, except for CMC.

### Toxicity test with ***C. elegans***

**Fig. 6:**
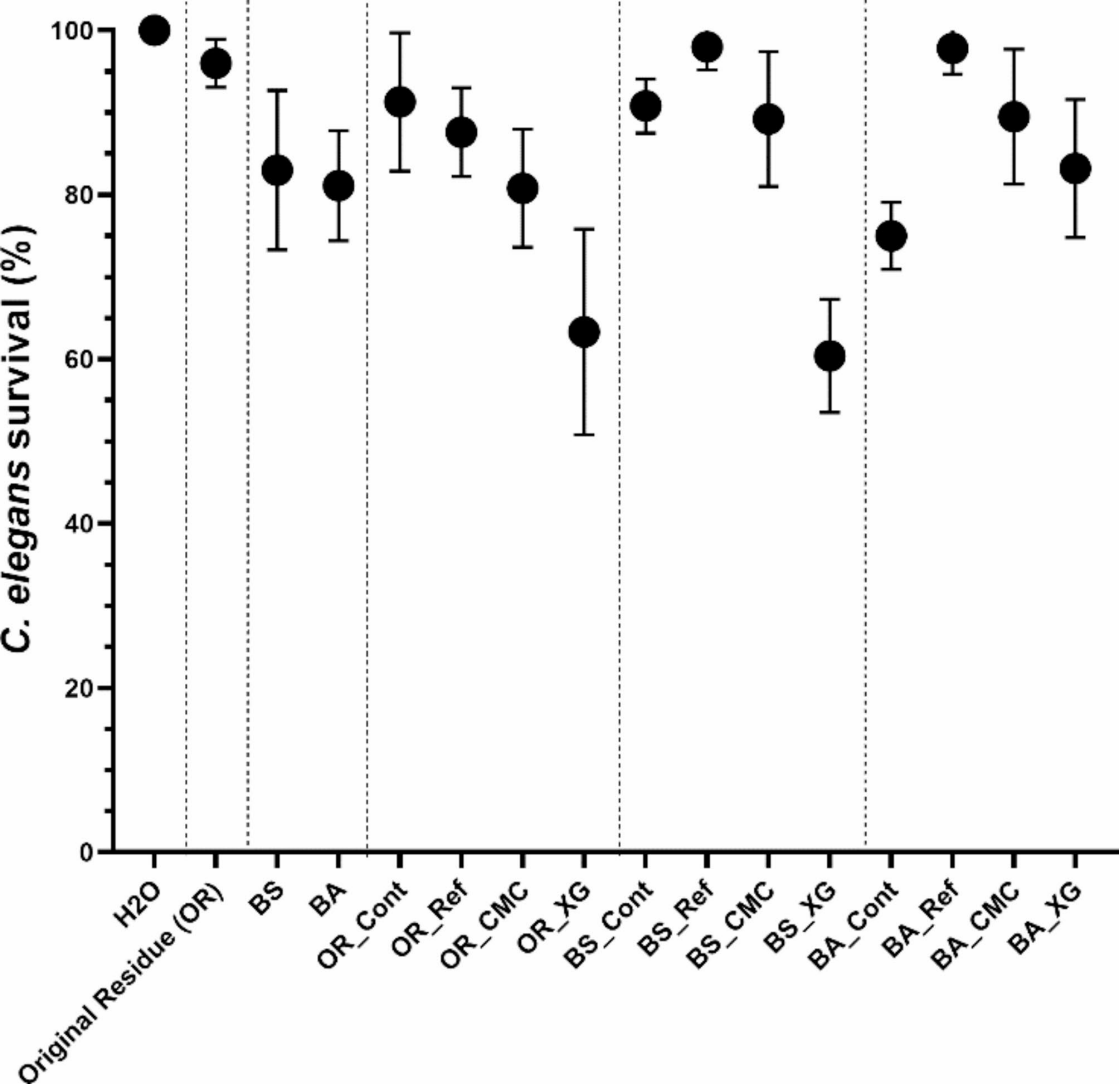
*C. elegans* survival (%) after exposure for 24 h to the residue-washed water. The data shown are the mean values of three independent experiments (± standard deviations).

The toxicity of the technosol was determined by studying its effect on *C. elegans*. The survival rate of nematodes was measured after they were exposed to water used to wash the residue and treated residues for 24 h. The supernatant from the original residue (OR) did not show relevant toxicity (Fig. 6), with over 95% of the nematodes surviving. When the residue was bioleached through biostimulation or bioaugmentation, it resulted in less than 20% mortality in *C. elegans*, a slight increase in mortality compared to the non-bioleached residue. Different stabilisers in the residue resulted in a supernatant with higher toxicity than the original residue (OR). The highest toxicity was observed with XG stabilisation (OR_XG − 36.7% ± 12.5% of mortality and BS_XG − 39.6% ± 6.9% of mortality). Regarding acute toxicity, the supernatants of the non-bioleached residue after stabilisation can be ranked as Ref > CMC > XG based on the increase in toxicity. Among the stabilised bioleached residues, the supernatant from cement (Ref) exhibited the greatest reduction in toxicity (almost zero toxicity), followed by CMC (approximately 85% of survival) and XG (varying between 63.3% ± 12.5% and 83.2% ± 8.4% of survival). Furthermore, when considering the effect of the two biotreatments independently, the stabilisation of bioleached residue with cement (Ref) and CMC reduced the leachates’ toxicity. The XG effect was dependent on the biotreatment.

### Analysis of the relative proportion of metals in solid samples after the different treatments

The solid samples were collected at the end of each experiment and analysed by XRF. The objective was to determine the relative proportion of metals in the residues. The variations indicate whether each metal was relatively enriched or depleted in the residue compared to the original residue (OR) (without any leaching cycle or treatment).

**Fig. 7:**
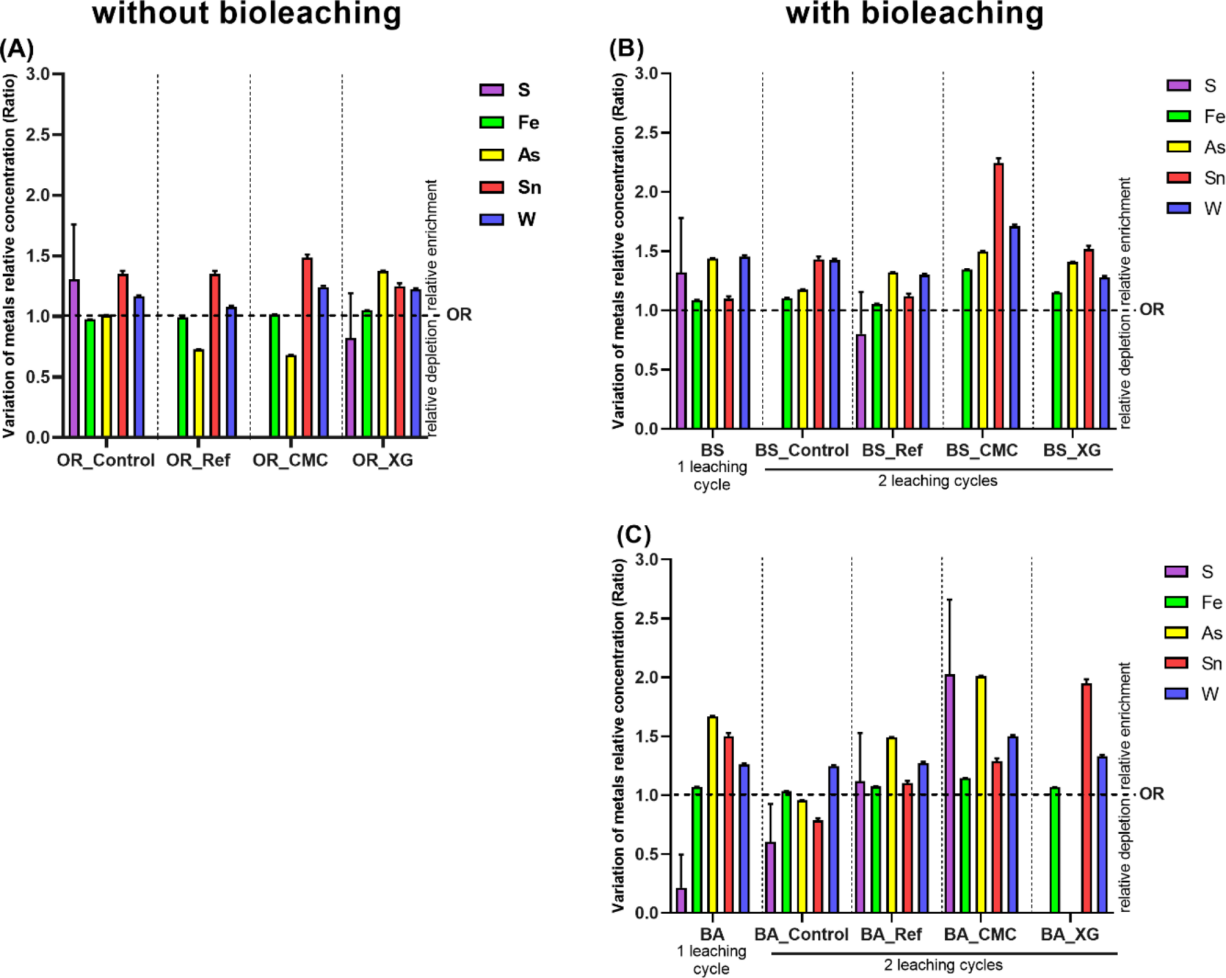
Variation of metals relative concentration ratio in the (**A**) residues without biotreatment (OR_Control, OR_Ref, OR_CMC and OR_XG), (**B**) residues biostimulated (with 1 leaching cycle BS and 2 leaching cycles BS_Control, BS_Ref, BS_CMC and BS_XG) and (**C**) residues bioaugmented (with 1 leaching cycle BA and 2 leaching cycles BA_Control, BA_Ref, BA_CMC and BA_XG).

Samples without biotreatment, stabilised or not (OR_Control, OR_Ref, OR_CMC and OR_XG) showed differences in the residue composition (Figs. 7A and S4). OR_Control showed an enrichment of S and tin (Sn) relative to the original residue (OR). Additionally, OR_Ref showed a relative depletion of S and As and a relative enrichment of Sn. OR_CMC had a relative enrichment of Sn and a relative depletion of S and As. OR_XG was relatively enriched with As and Sn.

After one leaching cycle, the biostimulated residue (BS) exhibits relative enrichment of Fe, K, Zn, and W compared to the original residue (OR) (Figs. 7B and S5). After stabilisation and the second leaching cycle, in general, the biostimulated residue exhibited greater differences in metal relative proportion when compared with residues that did not undergo biotreatment. The biostimulated residues (BS_Control, BS_Ref, BS_CMC and BS_XG) displayed a relative increase in Br, W and Pb compared to the original residue. After stabilisation, S was found only in BS_Ref. Compared with the original residue, the BS_CMC residue showed approximately a 2-fold relative enrichment of Sn. Furthermore, BS_CMC and BS_XG residues showed similar values for Sn.

Compared to the original residue, the bioaugmented residue with one leaching cycle (BA), showed the highest metal relative enrichments for As and Sn, and a visible relative depletion of S (Figs. 7C and S6). All bioaugmented residues had a relative enrichment of W, showing values of 1.1 to 1.7-fold higher than the original sample. The metal composition of the BA_Control residue, submitted to the second treatment without stabilisation, showed more similarity with the BA sample. When stabilised with cement (BA_Ref), this residue showed a similar composition to the BA sample. The CMC addition to the bioaugmented residue (BA_CMC) promoted a relative increase of S and As 2 times more than the original residue. The addition of XG (BA_XG) resulted in the total disappearance of S and As and doubled the relative concentration of Sn.

The metals highlighted in Fig. 7 were the most interesting. However, a more comprehensive analysis of the relative variation of metals can be possible with Figures S4, S5 and S6.

**Fig. 8:**
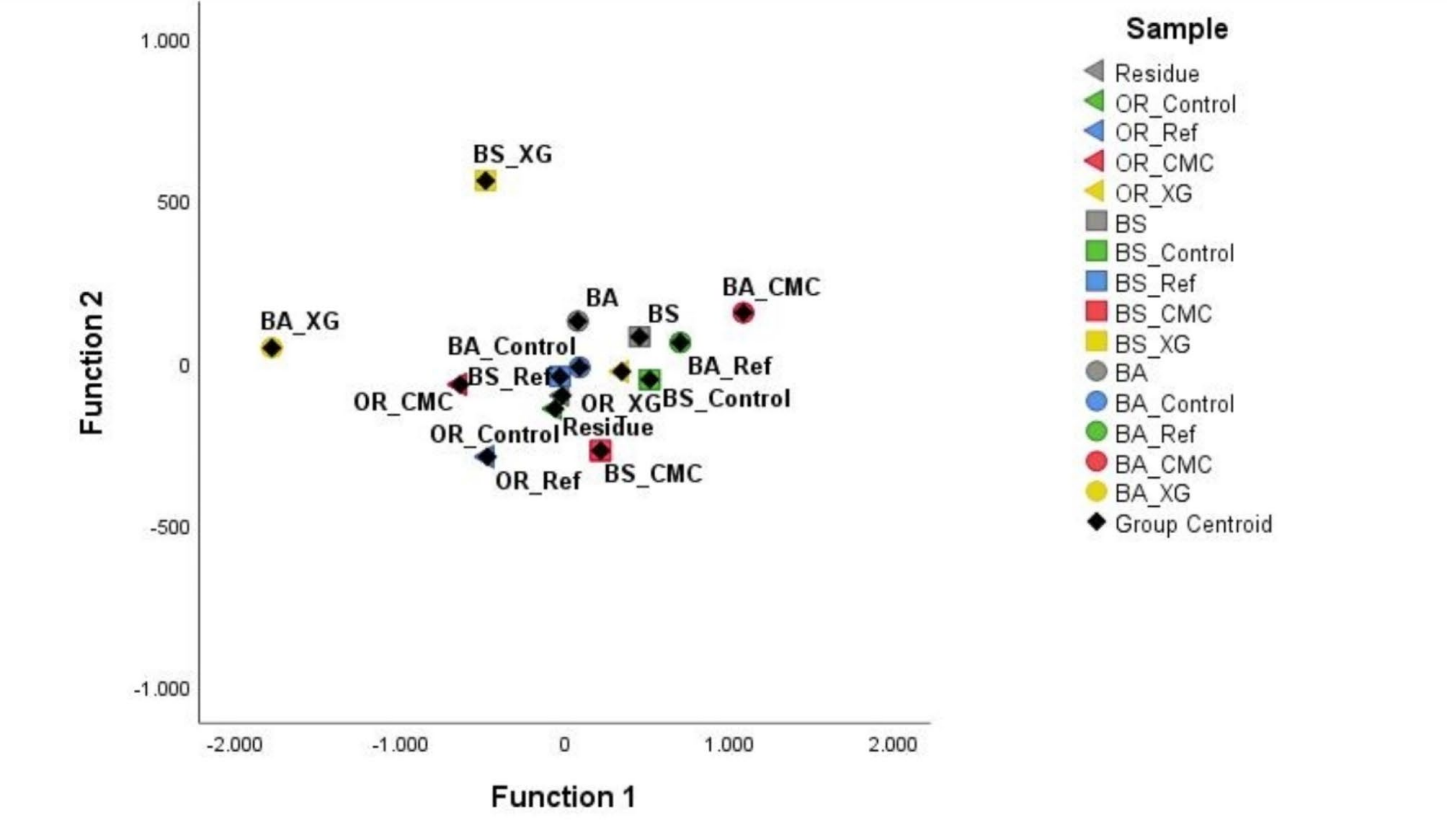
Leached residues discriminant function analysis based on metals concentration in all solid samples.

DFA was performed with the metal-relative concentrations of all solid samples (Fig. 8). The two Discriminant Functions explain, respectively, 86.6% (function 1) and 7.7% (function 2) of the variance (i.e., in Figs. 8 and 94.3% of the variance is explained). Function 1 was highly impacted by the relative concentration of zirconium (Zr) (7.953), yttrium (Y) (6.357), nickel (Ni) (5.983), S (5.107) and As (3.868) and negatively by the relative concentration of Sn (-6.129), strontium (Sr) (-3.495) and Co (-3.488). However, Function 2 was highly impacted by the relative concentration of Fe (3.194) and negatively by the relative concentration of Al (-1.604).

The separation of samples considering the stabiliser was not observed. However, both bioleached residues stabilised with XG (BS_XG and BA_XG) were the most distant samples, not grouped with the other residues.

## Discussion

The European Commission is concerned about the EU’s reliance on non-EU raw materials^33^. It is important to promote the utilisation of secondary sources, such as mine tailings as an alternative source of metals and other products to address this issue. The release of metals from soils poses a threat to some habitats, such as aquatic environments, and public health due to human exposure to heavy and toxic metals^34^. In this study, the leaching potential of residues was examined to assess the feasibility of using mine tailings (residue) as a secondary source of metals and test the release of the remaining metals during the production of material for technosol. Different biotreatments and biopolymers produced by bacteria were tested for their usefulness in treating the mine residues.

Previous research found that biostimulation of the residues leads to more metal leaching than bioaugmentation with strain B2A2W2 in this residue^5^. Similar results were observed in the present work, with the highest mobilisation observed for S and Fe (2.2-fold increase). Both biotreatments slightly raised the pH and biological activity of the residues, but bioaugmentation had a more significant effect (Tables 1, 3 and 4). The effect of the B2A2W2 bacterial strain on the bioaugmentation process is visible and, as more strains have been isolated from mining waste, it will be interesting to evaluate the effect of bioaugmentation with other strains. A study in Kaiping, China, showed that adding 0.5% w/w of CMC to the soil increased the number of microorganisms, due to CMC’s role as an organic substrate for microorganisms^35^. These findings align with the observed enhancement of CFU values with the different treatments, such as CMC stabilisation and bioaugmentation resulting in the highest number of viable cells. Total DNA molecular analysis would raise information on the dynamics of the residues microbiome. Biological activity in the soil helps to add organic matter, cycle nutrients, and create biodiversity. Increased soil biology provides food, optimal conditions, and minimal disturbance.

The leachate compositions were compared after two leaching cycles in this study. After the second cycle, the leachate content of the non-stabilised bioleached residue (BS_Control and BA_Control) was similar to that observed in the first (Table 1; Figs. 2 and 3). This suggests that not stabilised soils will continue to leach metal over time.

The presence of stabilisers exerted different effects on the residues not bioleached. Portland cement (OR_Ref) and CMC (OR_CMC) lead to an increase in the pH and CFU values, as reported in the literature^35,36^. The pH analysis of all samples showed that Portland cement raised the pH to a more neutral level, consistent with the literature^36^.

The choice of stabiliser also influenced the concentration of the metals in the leachate from bioleached residues (Figs. 2 and 3). When biostimulated, both Portland cement (BS_Ref) and CMC (BS_CMC) promoted S leaching in the first day of the experiment, while XG (BS_XG) promoted S, Fe and Zn leaching over time. Biopolymers CMC and XG promoted Fe leaching, which is not observed with cement. In the case of residues bioaugmented, the use of cement (BA_Ref) or CMC (BA_CMC) resulted in statistically lower Fe concentration in the leachates compared to XG (BA_XG). The leaching of Fe and other elements is observed in soils when the pH increases, and this is most likely one of the key factors altered by the use of different stabilisers^37^.

The results obtained from previous experiments showed that Portland cement and CMC had similar abilities to reduce the metal concentration in leachates. Studies concluded that CMC effectively immobilised heavy metals^34^, which is consistent with the results observed in this work (Figs. 2 and 3). Li and collaborators^34^ produced, with CMC, CMC-bridged chlorapatite nanoparticles which reduced 96–99% in Pb, Zn, Cu and Cd leaching from soil, corresponding to the immobilisation of these metals^30^. Exploring the effect of different concentrations of stabilizers could lead to more information on the role of the stabilizers on residue metal leaching.

A previous study found differences in the Panasqueira residue’s relative composition after the leaching process using XRF analysis^5^. Here, differences in the relative composition of the residues were also observed (Fig. 7). These differences corresponded to the enrichment in critical metals such as W and Sn. This relative enrichment is likely due to the high amount of S leached from the residue, which is naturally rich in S^26^. This effect was visibly enhanced by CMC stabilisation.

Fe and S were the elements most leached regardless of the treatment (Figs. 2 and 3). A study by Rito et al.^5^ revealed that Fe leaching increased over time when this residue was subject to water percolation. The time-dependent Fe mobilisation was related to the production of Fe^2+^ species at low pH. However, here, the observed increase in biological activity as reported by other studies, may justify the time-dependent Fe mobilisation^38^. As expected, the Fe leaching was particularly promoted by biotreatments and by the application of XG as a stabiliser, in our study. All samples stabilised with XG, regardless of being subjected or not to biotreatment, could leach high amounts of Fe that increased over time (higher than 99 mg × l^−1^ Fe).

Certain characteristics, such as element electronegativity or atomic radii^39,40^, can contribute to the easier leaching of Fe and S compared to critical metals like W and Sn. Additionally, the most noticeable differences in the composition of the treated residue compared to the original residue were observed in bioleached residues, highlighting the role of microorganisms in the leaching process. The use of different biopolymers is anticipated to result in the selection of distinct bacterial metabolic groups within the residue microbiome, impacting soil parameters, such as pH. A study on a Cu mine in Asia found a correlation between the presence of acidophilic bacteria (such as bacteria from the genera *Ferrovum*, *Firmicutes*, *Acidibacter*, and *Sulfobacillus*) with the use of Fe and S, leading to the mobilisation of metals^41^. Another experiment associated genes encoding proteins of the respiratory chain and iron oxidation (like iron-sulphur proteins) in bacteria that play a key role in acid mine drainages^42^. These studies show a link between the oxidation of Fe and S compounds and the presence of slow-growing microorganisms, such as *Thiobacillus* and *Acidithiobacillus* strains^41,42^. Additionally, in the column experiment study, the denitrification process and Fe(II) oxidation have been linked to the activity of denitrifying bacteria^43^. In the present work, the high amounts of Fe leached in bioaugmented samples may be explained by the presence of the denitrifying bacterium *D. polyhydroxybutyrativorans*^44^. Under the column conditions, Fe could have been used as an alternative electron donor for denitrification, mobilising it. The genome analysis of strain reveals several genes related to Fe, such as iron transport proteins (*feoAB* genes) and siderophores production (*exbB* gene)^45^.

Ecotoxicity tests were conducted using *C. elegans* (nematodes) due to their low-cost maintenance, easy manipulation with standard in vitro techniques and because 50% of *C. elegans* lethality is correlated with LC50 values obtained with other biological systems^46^. The original residue (OR) showed low acute toxicity, demonstrated by a high survival rate, while the biotreatments resulted in increased ecotoxicity of the residues (Fig. 6). It is important to note that almost all controls and residues stabilised with Portland cement showed low lethality, with survival values higher than 90%. When analysing the samples, only two residues (OR_XG and BS_XG) showed a survival rate considered moderately toxic (lethality higher than 30%) by some authors^47^. However, in comparison with results obtained with other mine soils, the acute toxicity values registered with Panasqueira mine residue, even after biotreatments and stabilisation, were low (results with mortality lower than 50%) and better than the previously published mine soils^48,49^. XG and CMC are biopolymers already used in some industries (like the food industry) and are described in the literature as not exhibiting toxicity^50^. However, residues’ stabilisation with XG increased the ecotoxicity of residues OR_XG and BS_XG, but not for the bioaugmented residue (BA_XG). The change in the ecotoxicity with XG might be related to the relative enrichment of As in these residues after (bio)treatments. However, BA_XG was the residue stabilised with XG that showed a higher relative depletion of As in the treated residue (Figs. 7 and S6). It is possible that in the presence of XG, strain B2A2W2 could have contributed to the difference in As mobilisation during bioleaching, since the genome analysis of the strain shows the presence of genes enabling the reduction and oxidation of As forms under regulation of two *arsR* genes (RAST Server)^45^. The low As in the BA_XG residues can be related to lower ecotoxicity (Fig. 6). This is according to previous results, where As is a potentially toxic element in soil that increases the hazard index concerning the health risk assessment of soils^51^.

Portland cement used had a concentration of 2.16% Mg oxide, which increased the amount of Mg in the samples. Samples stabilised with cement showed increased Mg content but not all the Mg was leached (Table 1; Figs. 2 and 3). Mg oxide has a significant impact on the resistance mechanisms to compression. Some authors linked the increase of soils’ compression strength with the hydration of active Mg oxide^52^. This implies that the leaching of Mg can affect the mechanical strength of the soil, justifying the decrease in compressive strength (Fig. 5) observed with Portland cement^15^. Nevertheless, the methodology used with leaching and homogenisation steps might have destroyed the bonds formed by cement particles, as mentioned in a previous study^15^.

The compressive strength obtained for leached samples was all higher than the obtained for samples not leached, except for CMC. This reflects the change in residues’ mineral composition. When comparing the compressive strength (Fig. 5) obtained for the samples without biotreatment and any leaching cycle, it is possible to observe that CMC resulted in the best performance, while XG was the worst stabiliser.

The samples after biotreatment showed similar behaviour in the UCS test (Fig. 5). XG performed best as a biopolymer stabiliser, while CMC was the worst. When comparing the two biotreatments, it was found that bioaugmentation resulted in higher values of UCS than biostimulation. The higher performance on compression strength with XG stabilisation was according to the described by Chang and collaborators^53^, who observed that XG increases the resistance of clay and sandy soils. The effect of XG in soils was previously related to its electric charge, promoting reactions between biopolymer molecules with water and soil particles^54^.

In conclusion, the stabilisers on non-bioleached residue promoted pH increase, different Fe concentrations leached, and similar soil’s compressive strength, except XG, which decreased the strength. Overall, CMC stabilisation led to an intermedia leaching profile, increased microbial biomass, and low ecotoxicity. On the other hand, XG stabilisation increased metal leaching and ecotoxicity of the residues and boosted the compressive strength with biotreatment.

When analysing the amount of metals, in the leachate and the residues, and the results of ecotoxicity, it is evident that the biotreatment and/or stabilisation of the Panasqueira mine residue led to the immobilisation of critical metals and a reduction in toxicity. Based on the results, this mine residue can be repurposed as a technosol after transformations. The residues biostimulated and stabilised with CMC produce a technosol suitable for bio-related applications, with low toxicity and reduced leaching. In terms of geotechnical applications, XG was found to be the most effective biopolymer stabiliser, enhancing compressive strength. An example of its application is in the construction sector, where it is used as a partial replacement of natural raw materials such as soils, sand, and gravel, which are applied in bases and sub-bases for communication infrastructures. Exploring bio-based processes for valuable metals recovery from secondary sources, like mine tailings, and the application of biopolymers as stabilisers of these residues can be beneficial in promoting alternative strategies and products that contribute to a more sustainable society.

## Methods

### Residue characterisation

The waste used in this work was collected from Basin 1 at Panasqueira mine, Fundão (Portugal), as described in previous work^26^, and stored at 4 °C. The residue was homogenised and air-dried naturally before measuring the water content. The optimal water content was established based on the Proctor compaction test^55^. The water content of the residue was modified to achieve the optimal Proctor value (19.5%).

### Sample preparation and bacteria growth

Each assay was prepared by introducing and compacting 160 g of residue into three layers inside a cylindrical PVC mould of 76 mm × 36 mm (height × diameter). Each layer was statically compacted with a steal bar 20 times and the surface of each one was sacrificed before the deposition of the next layer^56^.

Strain B2A2W2 was grown in R2A broth (R2Ab) medium for 48 h at 25 °C and 140 rpm. R2Ab medium contained per litre: 0.5 g of yeast extract, 0.5 g of proteose peptone, 0.5 g of casein, 0.5 g of glucose, 0.5 g of soluble starch, 0.3 g of dipotassium phosphate, 0.024 g of magnesium sulfate and 0.3 g of sodium pyruvate. The bacteria growth was centrifuged and the cellular pellets were washed three times with 0.85% NaCl solution and resuspended in R2Ab to obtain a bacterial suspension with an optical density at 600 nm (OD600) of approximately 1. The final bacterial suspensions were added to the residue for the top layer at a concentration of 5.0 × 10^7^ cells × g^−1^.

### Percolation system

**Fig. 9:**
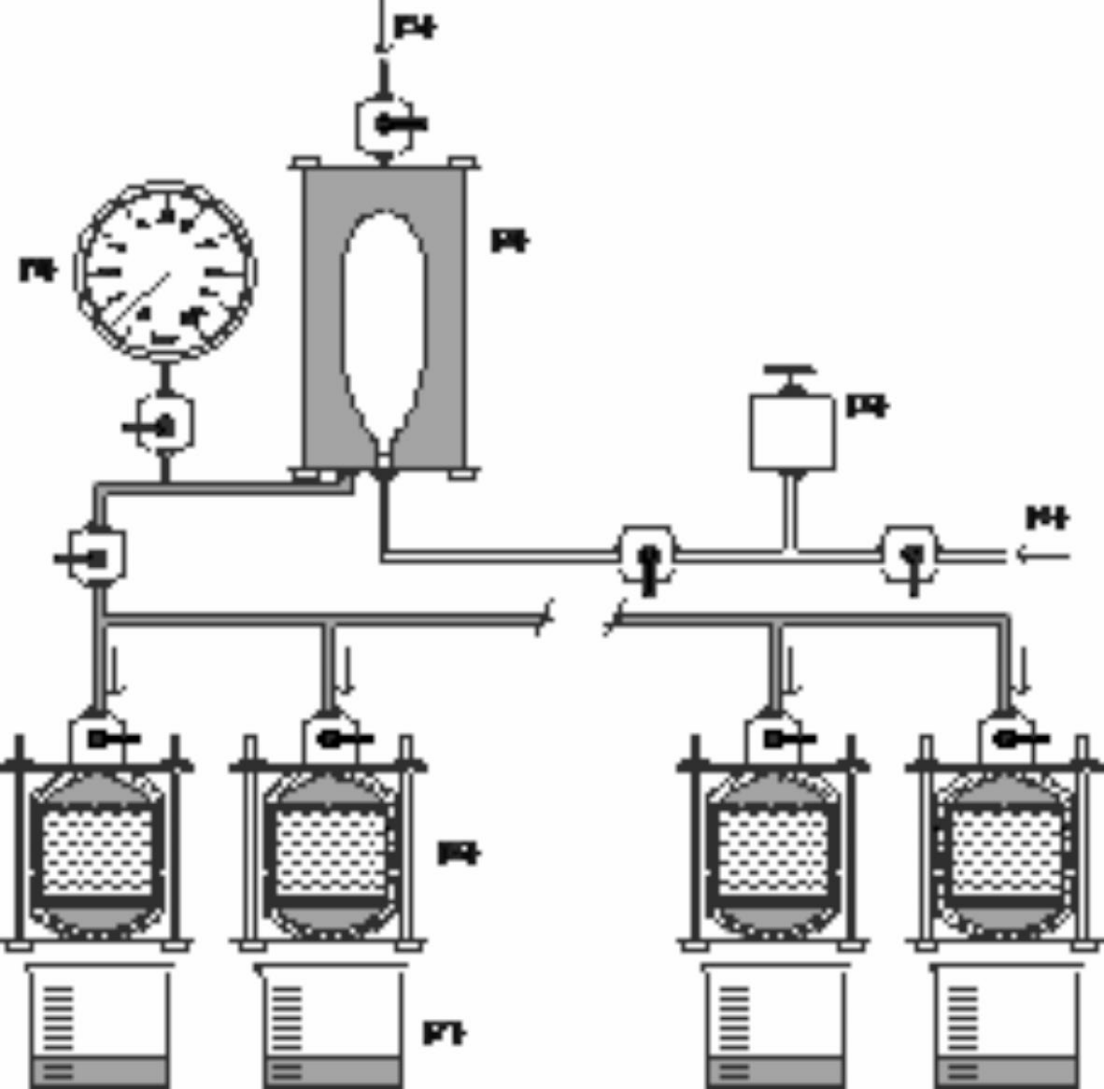
Percolation system scheme, composed of (**1**) pressure gauge, (**2**) air/liquid interface, (**3**) pressure regulator, (**4**) compressed air supply, (**5**) medium supply, (**6**) PVC columns and (**7**) effluent collectors (glass beakers).

Flow systems are essential for demonstrating the described concept and examining soil stabilisation by biopolymer application. The experimental flow system comprised a solution container (air/liquid interface), a PVC column, an effluent collector, and a constant-head permeability test with a pressure regulator, to control the liquid flow (Fig. 9). The samples in PVC moulds were inserted into the percolation system with a paper filter and perforated PVC disc, at both top and bottom to prevent the loss of solid particles. The experiments were performed at a pressure of 5 kPa, and the percolated liquid (leachate) was collected in 50 ml glass beakers. The system was constructed to test up to eight samples simultaneously and has already been used in previous work^15,56,57^. The experiments were conducted in a controlled environment at 20 ± 2 °C.

Before each assay, a disinfection procedure was conducted with a 50-fold diluted commercial bleach solution (sodium hypochlorite)^58^. Following the solution, the system was washed three times with distilled sterile water to remove sodium hypochlorite residues.

### Experimental design

**Fig. 10:**
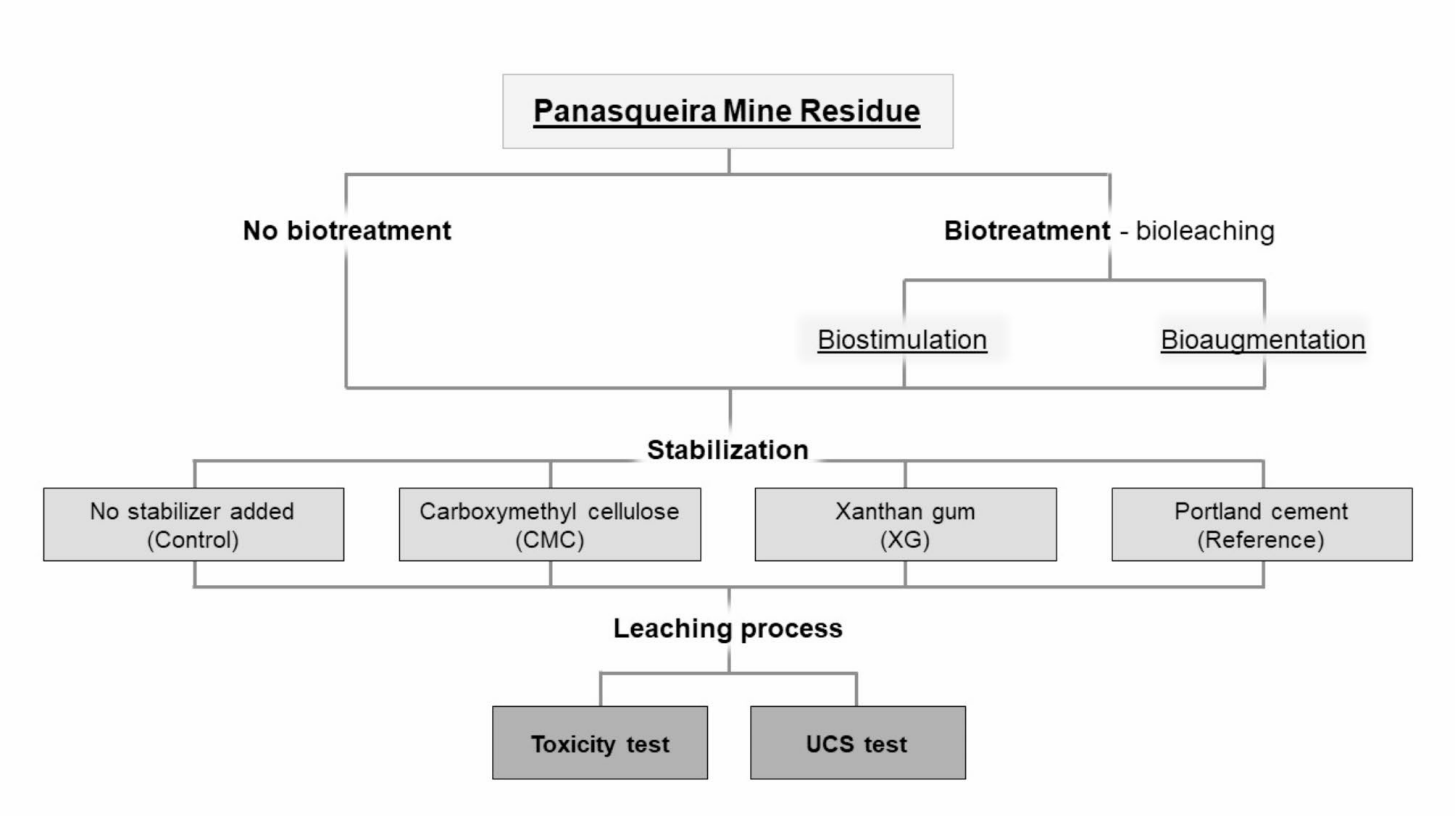
Flowchart of the experiments conducted.

Experiments were conducted to test different conditions to study the stabilisation of untreated and bioleached mine residue with biopolymers (Fig. 10). The bioleaching process was conducted in 7-day cycles. Firstly, the residue was bioleached by biostimulation with medium or bioaugmentation with the B2A2W2 strain. Then, the residues were stabilised with biopolymers CMC and XG, and Portland cement (as a reference) and cured. Subsequently, the third step consisted of the bioleaching process of residue-compound mixtures. Finally, the remaining residue-compound mixtures were cured for UCS tests.

### Bioleaching of residues

Mine residues were bioaugmented with B2A2W2 cell suspension or biostimulated with a 10-fold diluted R2Ab medium. The samples were identified as BA and BS (Table 5), respectively, and were subjected to the bioleaching cycle in the percolation system. The diluted R2Ab medium was used to flood the samples at different time points (0, 2, 5 and 7 days) and 20 ml of leachate was collected each time. R2Ab medium was selected due to its demonstrated efficacy in mobilising metals from the Panasqueira mine residue^5^. After the first leaching cycle (7 days), the leached waste was extracted from the PVC moulds, homogenised, and subsequently used. The 7 days were selected based on experimental results obtained with various bioleaching processes tested for Panasqueira mine residue^5^.

### Effect of the stabiliser addition on residues leaching and biological activity

The residues bioleached (described above) and residues non-bioleached were prepared without stabiliser addition (Control) or mixed with stabilisers: Portland cement (at a concentration of 1% w/w), CMC (1%) (Sigma-Aldrich) or XG (1%) (Sigma-Aldrich). The experiments were cured for 7 days in a controlled environment at 20 ± 2 °C and humidity of 95 to 100%^56^.

Samples were identified according to the stabilisers and the different residues used: non-bioleached (original residue—OR), bioleached by bioaugmentation (BA) and bioleached by biostimulation (BS) (Table 5). The residue-compound mixture was introduced into PVC moulds by the procedure previously described.

The leaching process started with the flooding of the samples with the 10-fold diluted R2Ab medium. The leachate was collected as previously described (0, 2, 5 and 7 days). At the end of each assay, 1 g of solid sample was collected for XRF analysis and toxicity tests. The remaining residue was removed from the PVC moulds, homogenised and reintroduced in the mould, in three compacted layers, and cured for an additional 7 days for the UCS test.

All leachate samples collected during both leaching cycles were analysed for pH and number of viable microorganisms. The microorganisms were analysed using the spread-plate technique on R2A solid medium (R2Ab medium with 15 g l^−1^ agar), incubated for 7 days at 25 °C and were expressed as CFU × ml^−1^. Samples from 0, 2 and 7 days were analysed by ICP-OES to measure the metal content.

**Table 5:** Summary of samples tested.

	Biotreatment	Number of leaching cycles	Stabiliser added
BS	Biostimulation	1	No stabiliser
BA	Bioaugmentation	1	No stabiliser
OR_Control	No biotreatment	1	No stabiliser
OR_Ref	No biotreatment	1	Portland cement
OR_CMC	No biotreatment	1	CMC
OR_XG	No biotreatment	1	XG
BS_Control	Biostimulation	2	No stabiliser
BS_Ref	Biostimulation	2	Portland cement
BS_CMC	Biostimulation	2	CMC
BS_XG	Biostimulation	2	XG
BA_Control	Bioaugmentation	2	No stabiliser
BA_Ref	Bioaugmentation	2	Portland cement
BA_CMC	Bioaugmentation	2	CMC
BA_XG	Bioaugmentation	2	XG

### UCS test

The UCS tests were conducted on a universal testing machine (Wykeham Farrance Tristar 5000 kg) under monotonic conditions, with a constant strain rate of 1% min^−1^. The stress–strain curves were analysed to determine the maximum unconfined compressive strength (q_u max_, kPa), the parameter used to characterise the mechanical behaviour in compression^59,60^. The UCS tests were performed on three different groups of samples: (1) residues without stabiliser or leaching cycle (control test); (2) residue-compound mixtures (residue with stabiliser) but without leaching cycles; and (3) residue-compound mixtures after leaching cycles. To ensure the reliability of the study, the tests were performed twice for each sample.

### Metals quantification

The metal quantification in the leachates (liquid) was performed by ICP-OES. Before analysis, the samples were filtered, acidified, and diluted with 2% nitric acid. The values obtained were analysed by calculating the absolute time-dependent variation. The variations were determined as the mean difference in each metal concentration between the final time (7 days) and the initial time (0 days) for each sample. The results are presented in this format to emphasise that different metals require different leaching times.

XRF was employed to determine the relative proportions of metals in the solid residues after leaching cycles. The resulting values were analysed by calculating the variation in metal concentration. This entailed calculating a ratio of the metal concentration in each sample and the metal concentration in the original residue.

### Informatics analysis

DFA was selected as the method for multivariate tests of differences between groups and to determine the probability of classifying the samples into these same groups. DFA was conducted using all variables and as a grouping variable the type of sample: OR_Control, OR_Ref, OR_CMC, OR_XG, BS, BS_Control, BS_Ref, BS_CMC, BS_XG, BA, BA_Control, BA_Ref, BA_CMC or BA_XG. The method was performed using the definitions of a covariance matrix within groups and all groups equal prior probabilities, as well as a combined-groups plot. DFA was performed using IBM SPSS Statistics for Windows, Version 25.0^61^.

Graphical figures and statistical analysis were obtained using GraphPad Prism version 9.3.1 for Windows^62^. The results analysed are the mean value of two or three independent experiments (the number of experiments is indicated in the figures’ captions) ± standard derivation. The statistical analysis was performed using two-way ANOVA followed by Tukey’s multiple comparisons test with a 95% confidence interval (α = 0.05).

### Toxicity test with ***C. elegans***

To test the acute toxicity of the residues obtained after the experiments, the residues were washed following ISO standards^63^. Each final residue (0.5 g) was washed with 10 ml of sterile deionised water at 150 rpm and 20 °C. After 24 h of incubation, samples were centrifuged at 5000 rpm for 20 min. The resulting supernatants were collected and filtered using 0.2 μm filters. The procedure was repeated a further seven times, with successive 24-h incubation periods^64^. The final supernatants obtained were tested for the survival of *C. elegans* exposed to the residue-washed water for 24 h^65^. The survival rate (%) was calculated as the ratio between the number of live *C. elegans* after 24 h and the number of live *C. elegans* at the beginning of the experiment.

## Electronic supplementary material

Below is the link to the electronic supplementary material.

Supplementary Material 1

## Data Availability

All data generated or analysed during this study are included in this published article (and its supplementary information files).
