# Supplementary material for: The effect of biopolymer stabilisation on biostimulated or bioaugmented mine residue for potential technosol production
Source: Sci Rep. 2024 Oct 26;14:25583. doi: 10.1038/s41598-024-75840-0 (PMC11513976; doi:10.1038/s41598-024-75840-0)
Supplement: Supplementary file 1 — Supplementary Material 1 [file 41598_2024_75840_MOESM1_ESM.docx]

**The effect of Biopolymer stabilisation on biostimulated or bioaugmented mine residue for potential technosol production**

**Joana B. Caldeira^1^, António A. Correia^2^, Rita Branco^1^, Paula V. Morais^1*^**

^1^ University of Coimbra, Centre for Mechanical Engineering, Materials and Processes, ARISE, Department of Life Sciences, 3000-456 Coimbra, Portugal

^2^ University of Coimbra, Chemical Engineering and Renewable Resources for Sustainability (CERES), Department of Civil Engineering, R. Luís Reis Santos, 3030-788 Coimbra, Portugal

*Corresponding author: pvmorais@uc.pt


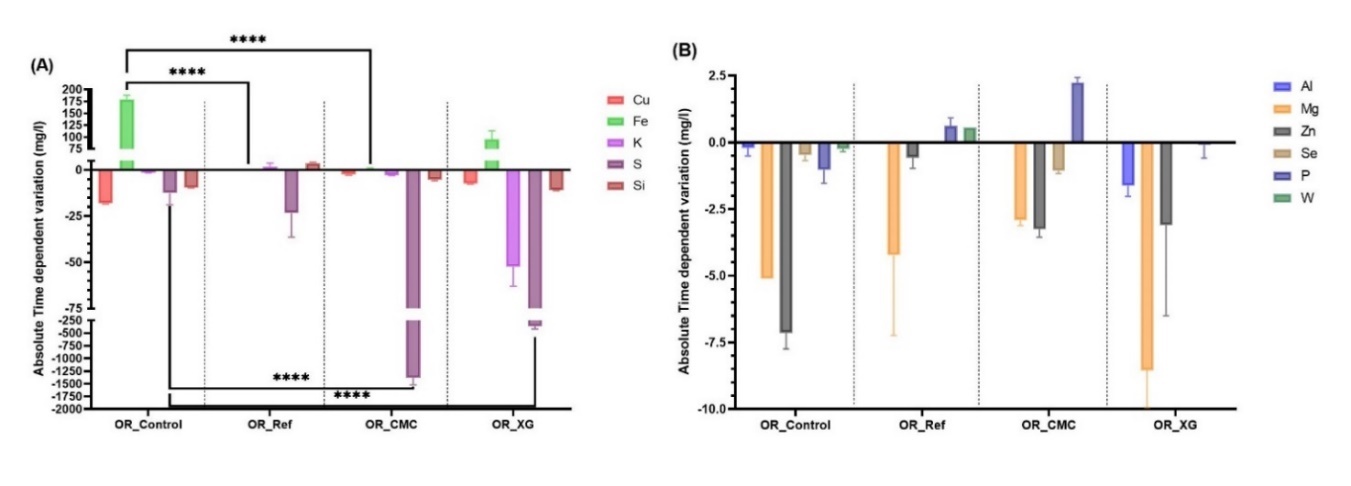


Figure S1. Absolute time-dependent variation of metal concentration (mg×l^-1^) in the leachates of residues, stabilised or not, OR_Control, OR_Ref, OR_CMC and OR_XG. Metals with high variation (> 10 mg×l^-1^) **(A)** and low variation (< 10 mg×l^-1^) **(B).** The variation was calculated from the difference in metal content between 7 d and 0 d. The data shown are the mean values of two independent experiments (± standard deviations). **** Significantly different, p < 0.0001.


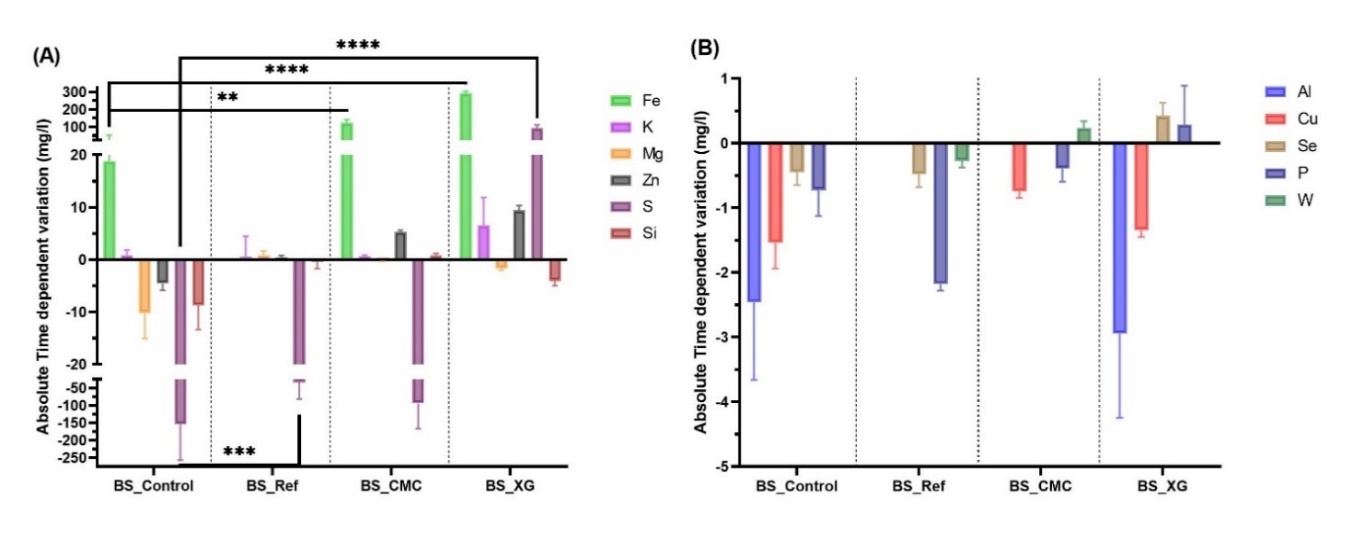


Figure S2. Second leaching cycle. Absolute time-dependent variation of metal concentration (mg×l^-1^) in the leachates of biostimulated residues, stabilised or not, BS_Control, BS_Ref, BS_CMC and BS_XG. Metals with high variation (> 5 mg×l^-1^) **(A)** and low variation (< 5 mg×l^-1^) **(B).** The variation was calculated from the difference in metal content between 7 d and 0 d. The data shown are the mean values of two independent experiments (± standard deviations). **,***,**** Significantly different, p < 0.01, p < 0.001 and p < 0.0001, respectively.


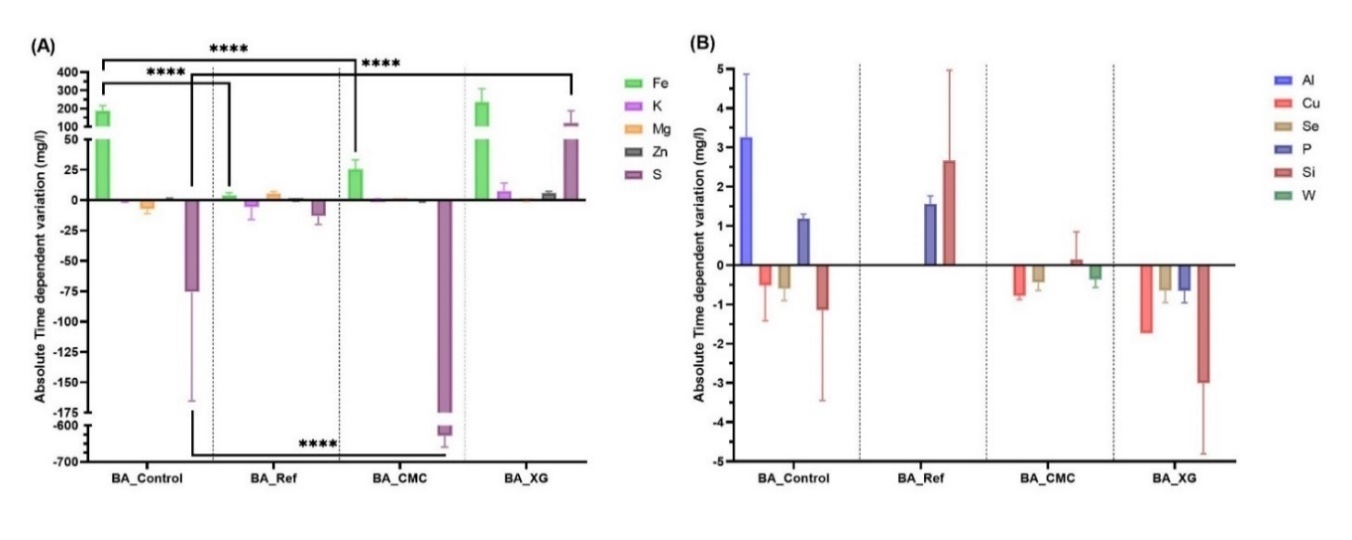


Figure S3. Second leaching cycle. Absolute time-dependent variation of metal concentration (mg×l^-1^) in the leachates of bioaugmented residues, stabilised or not, BA_Control, BA_Ref, BA_CMC and BA_XG. Metals with high variation (>5 mg×l^-1^) **(A)** and low variation (<5 mg×l^-1^) **(B).** The variation was calculated from the difference in metal content between 7 d and 0 d. The data shown are the mean values of two independent experiments (± standard deviations). **** Significantly different, p < 0.0001.


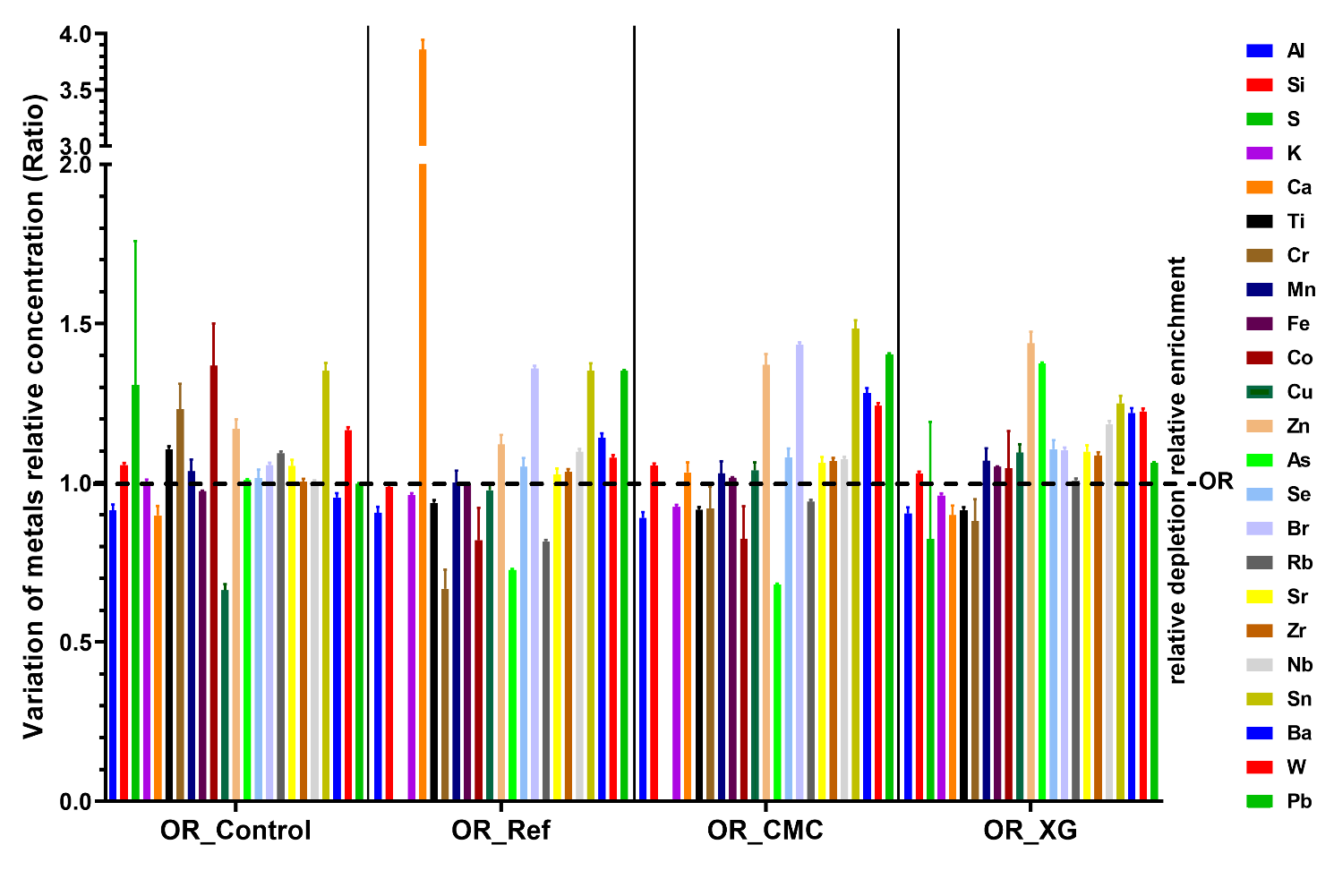


Figure S4. Variation of metals relative concentration (ratio) in the residues without biotreatment, stabilised or not (OR_Control, OR_Ref, OR_CMC and OR_XG).


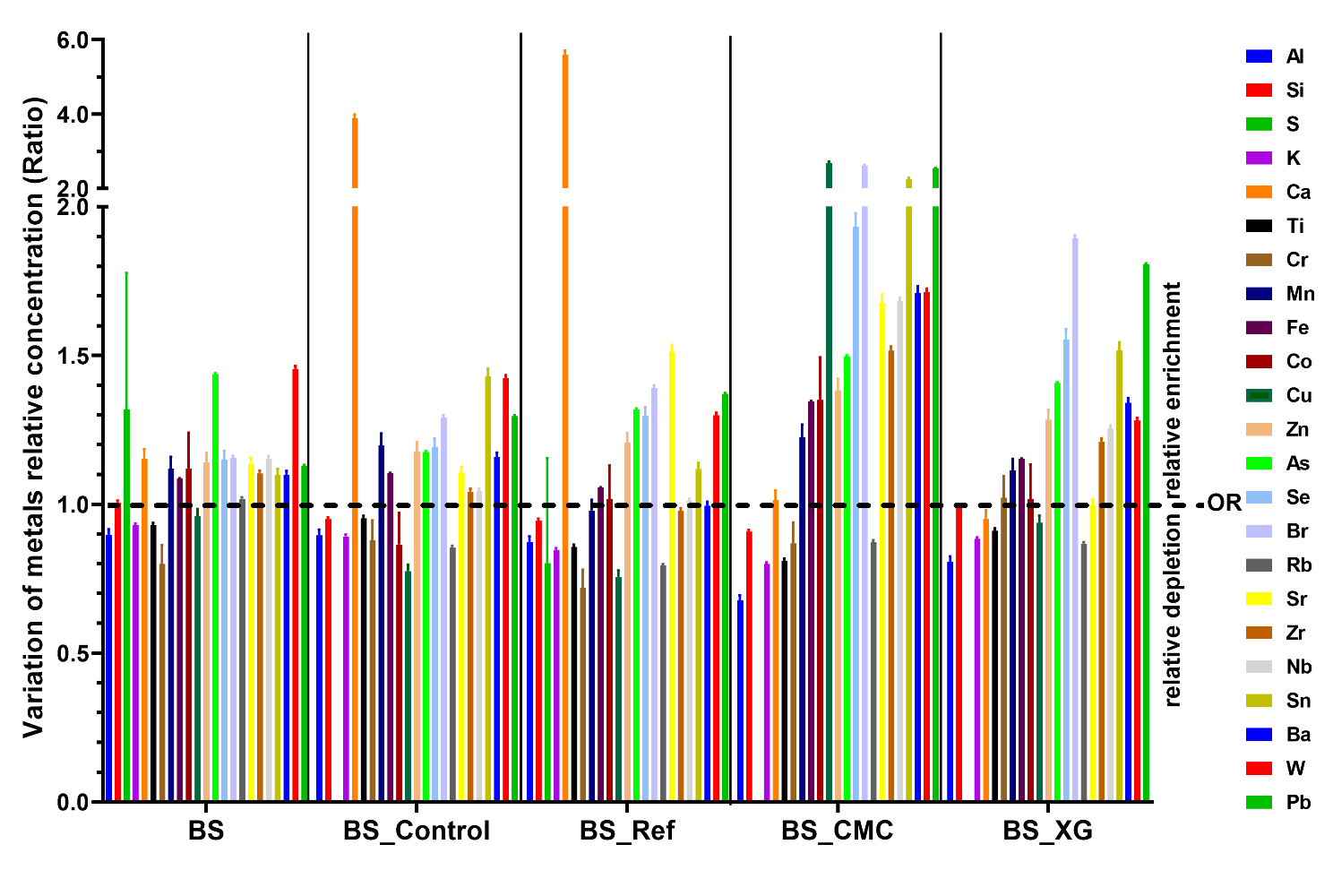


Figure S5. Variation of metals relative concentration (ratio) in the residues biostimulated, stabilised or not (BS_Control, BS_Ref, BS_CMC and BS_XG).


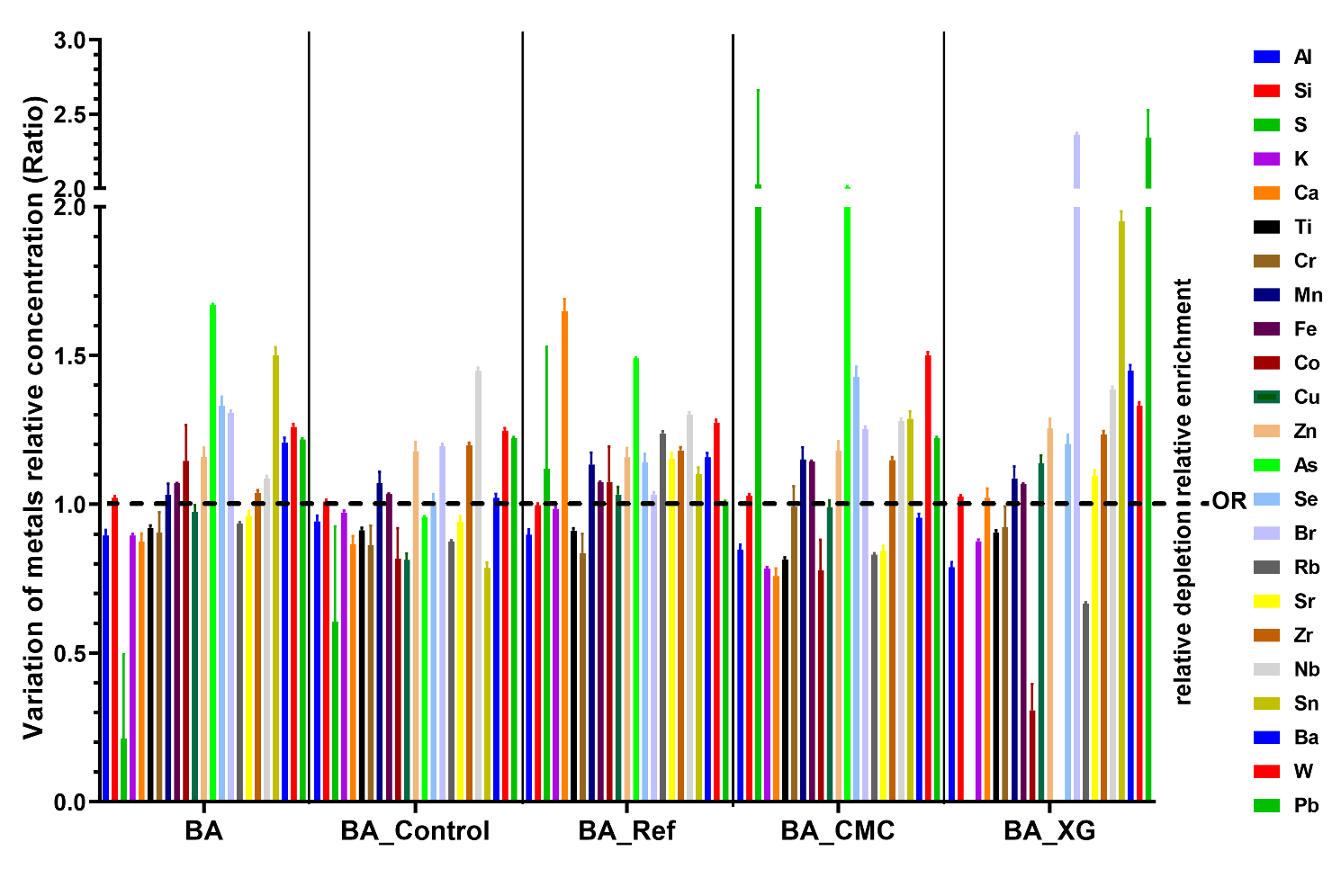


Figure S6. Variation of metals relative concentration (ratio) in the residues bioaugmented, stabilised or not (BA_Control, BA_Ref, BA_CMC and BA_XG).
